# From empirical efficacy to neurovascular and cerebrovascular systems perspective: a bibliometric and visualized analysis of traditional Chinese medicine for vascular dementia

**DOI:** 10.3389/fnut.2026.1880039

**Published:** 2026-10-02

**Authors:** Zixiang Jin, Qiuyue Huang, Xiaofen Jia, Miaomiao Lin, Jingwen Zhao, Jiahui Ren, Li Tang, Chenjie Huang, Yao Yang, Hui Ye, Xuezhi Zhang

**Affiliations:** 1 Department of Integrated Traditional Chinese and Western Medicine, Peking University First Hospital, Beijing, China; 2 Institute of Integrated Traditional Chinese and Western Medicine, Peking University, Beijing, China; 3 Xuanwu Hospital of Capital Medical University, Beijing, China; 4 Wangjing Hospital of China Academy of Chinese Medical Sciences, Beijing, China; 5 Peking University Third Hospital, Beijing, China; 6 Nanjing University of Chinese Medicine, Nanjing City, China

**Keywords:** bibliometric analysis, Chinese herbal medicine, acupuncture, chronic cerebral hypoperfusion, food–medicine homology, oxidative stress, traditional Chinese medicine, vascular dementia

## Abstract

**Objective:**

Vascular dementia (VaD) is a major form of cognitive impairment associated with cerebrovascular injury, neuroinflammation, oxidative stress, impaired neurovascular coupling, and progressive disruption of brain homeostasis. Traditional Chinese medicine (TCM)-related interventions for VaD encompass heterogeneous categories, including herbal formulas, single herbs, natural products, plant-derived bioactive compounds, acupuncture, electroacupuncture, and moxibustion. Within this broad landscape, the herbal and natural-product branch provides an important interface linking TCM research with food–medicine homology, neurological nutrition, and neurovascular protection. This bibliometric study aimed to map the research landscape, knowledge structure, hotspots, and emerging trends of TCM-related interventions for VaD, with particular attention to herbal, natural-product, and neurovascular research.

**Methods:**

English-language articles and reviews related to TCM treatment of VaD published from 1997 to 2025 were retrieved from the Web of Science Core Collection. VOSviewer, CiteSpace, and GraphPad Prism were used to analyze annual publication trends, countries/regions, institutions, authors, journals, co-cited references, and keywords. To address intervention heterogeneity, the findings were interpreted across herbal/natural-product, acupuncture/electroacupuncture, moxibustion, and mixed TCM-related categories.

**Results:**

A total of 357 publications were included, comprising 256 original articles and 101 reviews. China ranked first with 281 publications and occupied a central position in the collaboration network. High-frequency keywords included “vascular dementia,” “acupuncture,” “cognitive impairment,” “oxidative stress,” “apoptosis,” “hippocampus,” and “chronic cerebral hypoperfusion.” Two major research branches were identified. Herbal formulas, single herbs, natural products, and active compounds were mainly associated with oxidative stress, neuroinflammation, apoptosis, autophagy, chronic cerebral hypoperfusion, component characterization, and multi-target regulation. In contrast, acupuncture, electroacupuncture, and moxibustion were more closely related to neuromodulation, hippocampal plasticity, cerebral perfusion, and functional recovery. Emerging topics included network pharmacology, expression profiling, inflammasomes, component characterization, and mixed vascular–neurodegenerative mechanisms.

**Conclusion:**

The TCM-VaD literature represents a heterogeneous research landscape involving both herbal/natural-product and non-pharmacological intervention branches. Herbal medicines, natural products, and plant-derived bioactive compounds constitute the branch most directly connected with food–medicine homology, neurological nutrition, antioxidant and anti-inflammatory regulation, and neurovascular protection. However, bibliometric attention should not be interpreted as evidence of validated efficacy, and conclusions concerning phytoactive compounds should not be generalized to acupuncture- or moxibustion-related research. Future studies should strengthen intervention stratification, component standardization, mechanistic validation, and clinical translation.

## 1 Introduction

Vascular dementia (VaD) is a dementia-stage phenotype caused by or strongly associated with cerebrovascular disease and is commonly regarded as part of the broader spectrum of vascular cognitive impairment (VCI) and vascular cognitive impairment and dementia (VCID) (1, 2). VaD, VCI, and VCID are conceptually distinguished to avoid overgeneralization: VCI refers to a broad continuum of vascular-related cognitive impairment ranging from mild cognitive impairment to dementia; VCID is often used as an umbrella term covering vascular cognitive impairment and dementia; and VaD denotes the more severe dementia-stage condition. Because the present bibliometric dataset was constructed using VaD-related search terms, this study was designed to map TCM-related research specifically indexed around VaD, rather than the entire VCI/VCID field. Current evidence generally suggests that VaD accounts for approximately 15–20% of all dementia cases (2, 3). Broader epidemiological data on VCI/VCID are introduced here only to contextualize the disease burden of vascular-related cognitive impairment and should not be interpreted as indicating that the present dataset comprehensively covers post-stroke cognitive impairment, CSVD-related cognitive impairment, or mixed dementia. From the broader perspective of VCI/VCID, its disease burden is even more substantial, with the prevalence of post-stroke mild cognitive impairment and dementia estimated to be approximately 38 and 18.4%, respectively (4, 5). In China, the overall burden of Alzheimer’s disease and related dementias (ADRD) ranks among the highest worldwide. In 2019, the number of ADRD patients in China was approximately 13.14 million, accounting for about 25.5% of the global total, underscoring the major public health importance of preventing and managing cognitive disorders, including VaD and related vascular cognitive disorders (6, 7).

Unlike single-pathway neurodegenerative diseases, VaD is not driven by a single mechanism but represents a multilevel pathological syndrome involving vascular pathology, neural injury, and disruption of cognitive networks (1, 8). Cerebral small vessel disease (cSVD) is widely considered one of the most common pathological substrates of vascular cognitive impairment and may also contribute to VaD (8, 9). Its development and progression may involve upstream events such as chronic cerebral hypoperfusion, endothelial dysfunction, blood–brain barrier disruption, white matter hyperintensities, and lacunar injury (8–10), which may further lead to sustained cognitive decline through neuroinflammation, oxidative stress, mitochondrial energy dysfunction, impaired synaptic plasticity, and neuronal loss (1, 8). Meanwhile, increasing evidence indicates that vascular pathology and neurodegenerative pathology often coexist in older adults with cognitive impairment. However, these overlapping conditions are discussed in this study as related pathophysiological contexts rather than as independent bibliometric domains, because the search strategy did not independently target VCI, VCID, cSVD, post-stroke cognitive impairment, or mixed dementia (11, 12).

This complexity is reflected not only in etiological heterogeneity but also in marked temporal and spatial heterogeneity (4, 13–15). Temporally, vascular-related cognitive impairment may manifest as cognitive decline occurring shortly after stroke or as an insidious process arising from long-term accumulation of chronic hypoperfusion and cSVD-related injury; within this continuum, VaD represents the dementia-stage phenotype (4, 13, 15). Spatially, it involves region-specific damage affecting the hippocampus, subcortical white matter, deep gray nuclei, and the neurovascular unit interface (14, 16, 17). Accordingly, current VaD research is no longer confined to the traditional classification of dementia subtypes but is gradually shifting toward an integrated framework emphasizing pathological stratification, early identification, and intervenable mechanisms (8). In the present manuscript, such concepts are used to help interpret the mechanistic background and future research directions of VaD-focused TCM studies, but the bibliometric findings should not be generalized to the entire VCI/VCID, cSVD, or mixed dementia literature. Recent studies have further suggested that, beyond classical inflammatory and oxidative stress mechanisms, emerging biophysical processes such as proteostasis imbalance, biomolecular condensates, and liquid–liquid phase separation may offer new theoretical perspectives for understanding the intersection between vascular injury and neurodegenerative pathology (18, 19).

Against this background, identifying therapeutic strategies capable of targeting multiple pathological processes while integrating holistic regulation with mechanism-based intervention has become an important direction in VaD research (20). Traditional Chinese medicine (TCM) has a long history of application in the prevention and treatment of cognitive disorders and brain diseases. Related studies have developed multiple forms of intervention, including herbal formulas, single herbs, natural products, active compounds, acupuncture, and moxibustion (21, 22). Within this heterogeneous TCM landscape, herbal formulas, single herbs, natural products, and isolated bioactive compounds constitute the branch most directly connected with plant-derived neuroprotective research. In particular, the modern concept of food–medicine homology extends beyond the traditional overlap between medicinal and edible materials and increasingly emphasizes functional constituents, processing, health maintenance, safety, and translational evaluation (23). This framework provides a relevant nutritional and pharmacological context for interpreting medicinal and edible plant resources, phytoactive compounds, and multi-component herbal interventions in neurological health. Nevertheless, food–medicine homologous resources represent only one component of TCM-related VaD research, and these intervention categories should not be regarded as biologically equivalent. Chinese herbal formulas are usually based on multi-component pharmacology and TCM prescription logic; single herbs, extracts, natural products, and isolated active compounds are more closely related to pharmacological material basis and lead-compound research; recent reviews on food–medicine homologous Chinese medicines from Leguminosae species also suggest that plant-derived bioactive constituents may be discussed within a neuroprotective framework, although such evidence should still be interpreted according to specific disease contexts and validation levels (24); acupuncture and electroacupuncture are non-pharmacological interventions involving neuromodulatory, somatosensory, and sensory-vascular mechanisms; and moxibustion additionally involves thermal stimulation and local/systemic regulatory effects. Accordingly, natural products and phytoactive compounds in this manuscript are discussed as one research branch of TCM-related VaD studies, mainly involving material basis exploration, pharmacological hypotheses, and preclinical candidate mechanisms. They should not be interpreted as representing the entire TCM-VaD field, nor should bibliometric attention to these terms be regarded as evidence of clinical validation. Therefore, in this manuscript, “TCM-related interventions” is used as an umbrella term, while the major intervention categories are interpreted separately whenever possible. From a clinical perspective, these different intervention categories also require distinct evidence standards. Herbal formulas require adequate prescription rationale, quality control, dose standardization, and safety monitoring, whereas acupuncture, electroacupuncture, and moxibustion require standardized reporting of acupoint selection, stimulation parameters, treatment frequency, practitioner-related factors, sham or active comparator design, and blinding feasibility. Therefore, clinical relevance in this field should be interpreted not only according to publication volume or keyword frequency, but also according to diagnostic consistency, trial design, outcome selection, intervention reproducibility, follow-up duration, and safety reporting. Research has gradually expanded from empirical efficacy observations to mechanistic investigations involving inflammation, oxidative stress, apoptosis, autophagy regulation, neuroregeneration, neurovascular unit homeostasis, and brain functional remodeling. With advances in analytical chemistry, systems pharmacology, and multi-omics technologies, research on TCM for VaD is shifting from conventional efficacy evaluation toward component characterization, interaction network analysis, and the coupling of high-dimensional neurovascular biological mechanisms (22, 25).

However, although a substantial body of literature has accumulated in basic experiments, clinical observations, systematic reviews, and mechanistic studies, systematic mapping of the overall knowledge structure, major research forces, intellectual foundations, hotspot themes, and frontier evolution of this field remains insufficient. Compared with traditional reviews, bibliometric analysis enables macroscopic visualization of publication trends, country and institutional distributions, author collaboration networks, journal influence, co-cited knowledge bases, and keyword evolution pathways, thereby providing a clearer understanding of research trajectories, hotspot concentrations, and future directions (26, 27).

Therefore, based on the Web of Science Core Collection (WoSCC) database, this study used VOSviewer and CiteSpace to conduct a bibliometric and visualized analysis of publications related to TCM treatment of VaD from 1997 to 2025. This study should therefore be understood as a VaD-centered bibliometric and science-mapping review. Although VCI/VCID, cSVD, post-stroke cognitive impairment, and mixed dementia are discussed as related clinical and pathophysiological contexts, they were not used as expanded disease-search categories in the present analysis. The aim was to systematically reveal the research landscape, intellectual structure, hotspot themes, and developmental trends in this field, with particular attention to herbal, natural-product, and neurovascular research, while distinguishing these themes from acupuncture- and moxibustion-related research.

## 2 Materials and methods

### 2.1 Data source and search strategy

WoSCC is widely recognized as an important data source for bibliometric studies because of its high-quality literature coverage, complete citation information, and standardized document-type classification. Therefore, WoSCC was selected as the data source for this study (28). WoSCC was used as the only database for the main bibliometric analysis to maintain consistency in citation data structure, document-type classification, and export format. The search was completed on March 20, 2026, and covered publications from database inception to December 31, 2025. The search topic focused on studies related to TCM treatment of VaD. It should be noted that the disease-related component of the search strategy was intentionally restricted to VaD-related terms. Terms such as VCI, VCID, post-stroke cognitive impairment, cerebral small vessel disease, and mixed dementia were not included as independent search categories; therefore, the resulting dataset reflects a VaD-centered literature corpus rather than the entire vascular cognitive impairment research field.

The search strategy was designed to balance retrieval sensitivity and thematic specificity. The TCM-related search terms included both core TCM terms, such as “traditional Chinese medicine,” “TCM,” “Chinese herb,” “acupuncture,” and “moxibustion,” and broader plant-derived or natural product terms, such as “natural product,” “natural compound,” “phytochemical,” “secondary metabolite,” “natural medicine,” and “herb.” These broader terms were retained because many studies on Chinese herbal medicines, active herbal constituents, and plant-derived bioactive compounds may be indexed in WoSCC under natural product or phytochemical terminology rather than explicitly under “traditional Chinese medicine.” However, these broad terms were not used to define all natural product studies as TCM-related. Instead, they were used only as retrieval terms, and all records were subsequently screened manually according to predefined eligibility criteria to ensure direct relevance to TCM-related interventions for VaD. Before data export, the Boolean structure of the search query was carefully checked in the WoSCC Advanced Search interface. The TCM-related term set and the VaD-related term set were combined using the intended parenthetical grouping, so that all TCM-related terms were searched in combination with the VaD-related terms. To improve transparency and reproducibility, the query below is presented as the exact WoSCC topic search query used for data retrieval, rather than a reconstructed description in prose.

The exact WoSCC topic search query was as follows:

((((((((((TS = (“natural product*”)) OR TS = (“natural compound*”)) OR TS = (“traditional Chinese medicine*”)) OR TS = (TCM*)) OR TS = (“Chinese herb*”)) OR TS = (“phytochemical*”)) OR TS = (“secondary metabolite*”)) OR TS = (“natural medicine*”)) OR TS = (“Herb*”)) OR TS = (“acupuncture”)) OR TS = (“moxibustion”)) AND ((((TS = (“Vascular Dementias”)) OR TS = (“Vascular Dementia”)) OR TS = (“Arteriosclerotic Dementia*”)) OR TS = (“Subcortical Vascular Dementia”)) OR TS = (“Acute Onset Vascular Dementia”)). The complete retrieval records were exported from WoSCC after applying the intended Boolean grouping, language restriction, and document-type restriction. The initial WoSCC search retrieved 384 records. Records were then screened sequentially according to publication year, document type, language, and duplication status. Nine records published outside the predefined period of 1997–2025 were excluded, leaving 375 records. After limiting document types to Articles and Reviews, 359 records remained. Two non-English records were further excluded. Duplicate checking was performed using CiteSpace, and no duplicate records were identified. Finally, 357 publications were included in the bibliometric analysis. All eligible records were exported as plain text files in the “Full Record and Cited References” format for subsequent bibliometric analysis.

### 2.2 Inclusion and exclusion criteria

To improve the transparency and reproducibility of the screening process, the retrieved records were screened according to predefined bibliometric eligibility criteria. The screening process was conducted sequentially according to publication year, document type, language, and duplication status. Two reviewers independently checked the exported records and verified whether the final records were consistent with the predefined topic and eligibility criteria. Any disagreements were resolved through discussion and consensus; when necessary, a third senior reviewer was consulted. Inter-rater agreement was not formally assessed.

The inclusion criteria were as follows:

1. Studies related to TCM treatment of VaD, including basic research, clinical studies, or review articles involving TCM-related interventions such as Chinese herbal medicine, natural products, acupuncture, and moxibustion.
2. Document types limited to Articles or Reviews.
3. Publications written in English.
4. Publications published between 1997 and 2025.
5. Records exported from WoSCC with complete bibliographic information and cited references.

The exclusion criteria were as follows:

1. Records published outside the predefined period of 1997–2025.
2. Document types other than Articles or Reviews, including editorial materials, letters, news items, meeting abstracts, corrections, proceedings papers, and book chapters.
3. Non-English publications.
4. Duplicate records identified during CiteSpace duplicate checking. No duplicate records were identified in this study. The literature retrieval and screening process is shown in Figure 1.

**Figure 1:**
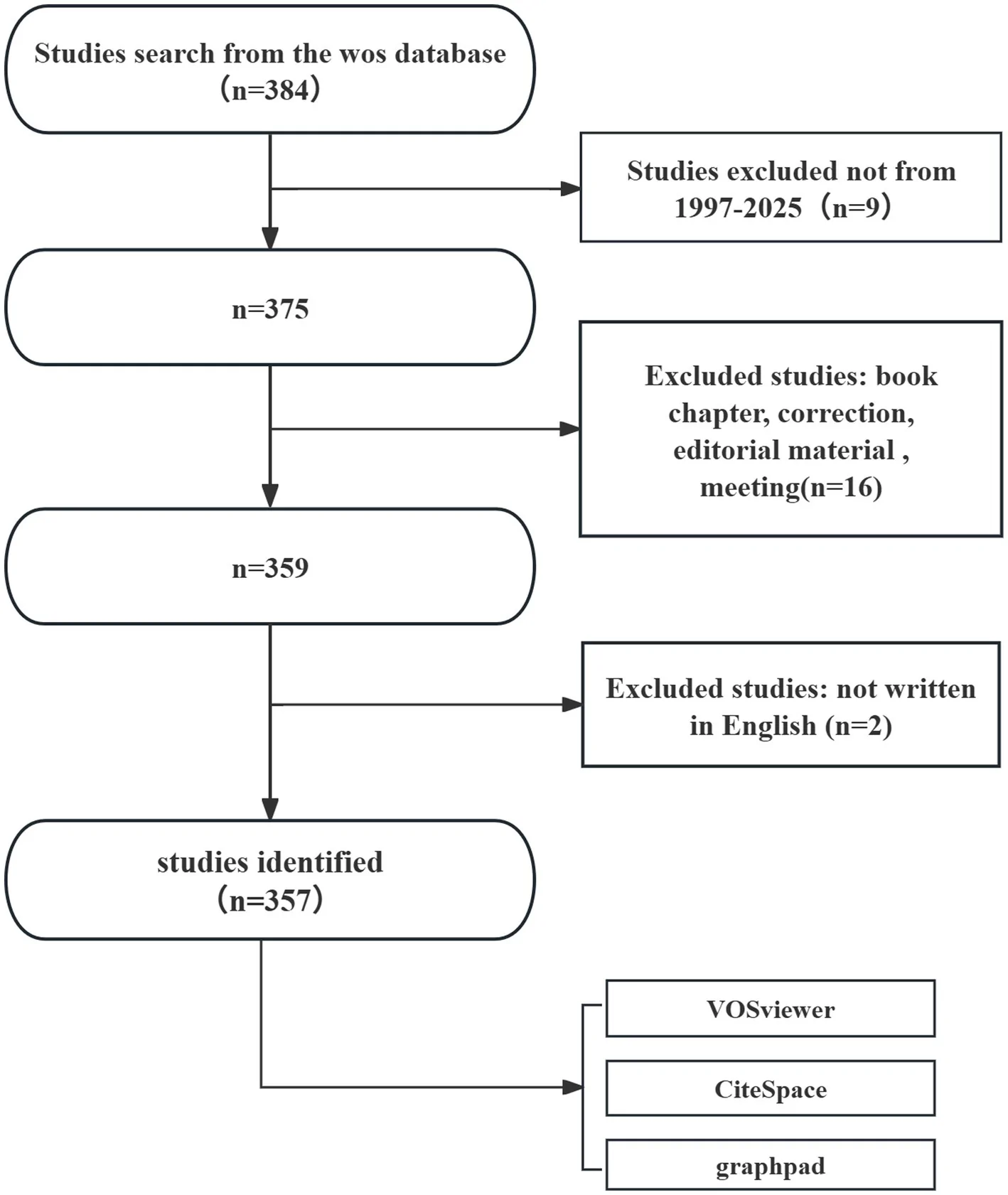
Flow diagram of literature retrieval and screening.

### 2.3 Data extraction and bibliometric analysis

All retrieved records were exported in plain text format, including complete bibliographic information and cited references. Journal Impact Factor and quartile information for the journals listed in Tables 1, 2 were obtained from Clarivate Journal Citation Reports. Specifically, the journal indicators were based on the 2025 Journal Citation Reports release, which reports 2024 JCR data. Therefore, journal impact indicators are reported as the 2024 Journal Impact Factor and corresponding JCR quartile based on the JCR 2025 release. GraphPad Prism 8.0.2 was used to plot annual publication trends and country-level publication trends. VOSviewer (version 1.6.18) (29) was used to construct co-occurrence networks of countries, institutions, authors, journals, and keywords. For VOSviewer analysis, the full counting method was used, and association strength normalization was applied to construct and visualize bibliometric networks. The minimum thresholds were set according to the specific network type: countries/regions with at least 1 publication, institutions with at least 2 publications, authors with at least 2 publications, journals with at least 2 publications, and keywords with at least 5 occurrences were included in the corresponding maps. Before keyword co-occurrence analysis, keyword cleaning and harmonization were performed manually by two reviewers to reduce spelling variation, abbreviation inconsistency, and semantic duplication. First, synonyms, abbreviations, and variant spellings were standardized; for example, “VaD,” “VD,” and “vascular dementia” were merged as “vascular dementia,” and “traditional Chinese medicine,” “TCM,” and related spelling variants were standardized as “traditional Chinese medicine.” Second, terms with the same meaning but different written forms were merged, such as “NF-kappa B” and “NF-κB,” and “electro-acupuncture” and “electroacupuncture.” Third, overly nonspecific bibliographic terms that did not contribute to thematic interpretation were removed when appropriate. However, some broad but high-frequency terms, such as “brain,” “model,” “mechanisms,” and “impairment,” were retained in the frequency table when they met the occurrence threshold, because they reflected common indexing patterns in the original WoSCC records. These broad terms were interpreted cautiously and were not regarded as independent mechanistic themes. In addition, “cognitive impairment” and “cognitive function” were not merged because the former primarily denotes a pathological or clinical state, whereas the latter is commonly used as an outcome-related term in intervention studies. For co-occurrence analysis, nonspecific and semantically duplicated keywords were manually cleaned and merged before network construction. VOSviewer’s default clustering algorithm was used, with the resolution parameter set to 1.00 and the minimum cluster size set to 1. CiteSpace (version 6.2.4R) (30) was used separately to analyze co-cited references, temporal keyword clustering, burst detection, and the dual-map overlay of journals. Keyword clustering outputs generated by VOSviewer and CiteSpace were treated as separate analytical products because the two programs use different clustering algorithms and visualization logic; therefore, no one-to-one correspondence between their clusters was assumed. Through integrated analysis using multiple software tools, this study systematically revealed the research landscape, hotspot themes, and evolutionary trends in the field of TCM treatment for VaD. VOSviewer, developed by Waltman et al. in 2009, is a free Java-based software tool used to analyze large-scale bibliographic data and visualize the results in map-based formats, particularly for constructing and displaying co-citation networks (31). CiteSpace, developed by Professor Chaomei Chen, was also designed within an experimental framework for detecting emerging concepts and evaluating existing scientific knowledge structures. It enables users to better understand knowledge domains, research frontiers, and developmental trends, and to predict future research directions (32). Similar VOSviewer- and CiteSpace-based visualized analyses have also been applied to traditional Chinese medicine resource research, such as licorice-related research hotspots and trends, supporting the applicability of knowledge-map methods in TCM resource-oriented fields (33). To address the heterogeneity of TCM-related interventions, the included studies and keyword themes were further interpreted according to broad intervention categories where possible: (1) herbal formulas and classical prescriptions; (2) single herbs, herbal extracts, natural products, and isolated active compounds; (3) acupuncture and electroacupuncture; (4) moxibustion; and (5) mixed or unspecified TCM-related interventions. This stratified interpretation was used to avoid presenting all TCM-related studies as a single homogeneous intervention category. In particular, herbal/natural product studies and acupuncture/moxibustion studies were considered separately when interpreting keyword clusters and research hotspots. Food–medicine homology and neurological nutrition were used only as interpretive contexts for the herbal and natural-product branch, rather than as independent retrieval categories or descriptors of the entire TCM-VaD literature.

**Table 1:** Journal publication output.

Rank	Journal	Article counts	Percentage	IF	Quartile in category
1	Evidence-Based Complementary and Alternative Medicine	22	6.16%	2.6	Q2
2	Journal of Ethnopharmacology	20	5.60%	5.4	Q1
3	Neural Regeneration Research	15	4.20%	6.7	Q1
4	Medicine	14	3.92%	1.4	Q2
5	Frontiers in Pharmacology	11	3.08%	4.8	Q1
6	Frontiers in Aging Neuroscience	9	2.52%	4.5	Q1
7	Phytomedicine	9	2.52%	8.3	Q1
8	BMC Complementary Medicine and Therapies	7	1.96%	4.7	Q1
9	Phytotherapy Research	7	1.96%	6.3	Q1
10	Frontiers in Neuroscience	6	1.68%	3.2	Q2

**Table 2:** Co-cited journals.

Rank	Cited journal	Co-citation	2024 Journal impact factor (JCR 2025 release)	Quartile in category
1	Stroke	169	8.9	Q1
2	Journal of Ethnopharmacology	141	5.4	Q1
3	Evidence-Based Complementary and Alternative Medicine	138	2.6	Q2
4	Brain Research	134	2.6	Q3
5	PLOS ONE	134	2.4	Q2
6	Journal of Alzheimer’s Disease	124	3.1	Q2
7	The Lancet	123	88.5	Q1
8	Neuroscience Letters	119	2	Q4
9	Neural Regeneration Research	101	6.7	Q1
10	International Journal of Molecular Sciences	100	4.9	Q1

## 3 Results

### 3.1 Overall characteristics of the literature and annual publication trends

Figure 2A presents the annual publication trends in studies related to TCM treatment of VaD from 1997 to 2025. According to the search strategy, a total of 357 publications related to TCM treatment of VaD were retrieved from the Web of Science Core Collection (WoSCC), including 256 original articles and 101 reviews. These studies involved 34 countries/regions, 475 institutions, and 1,758 authors.

**Figure 2:**
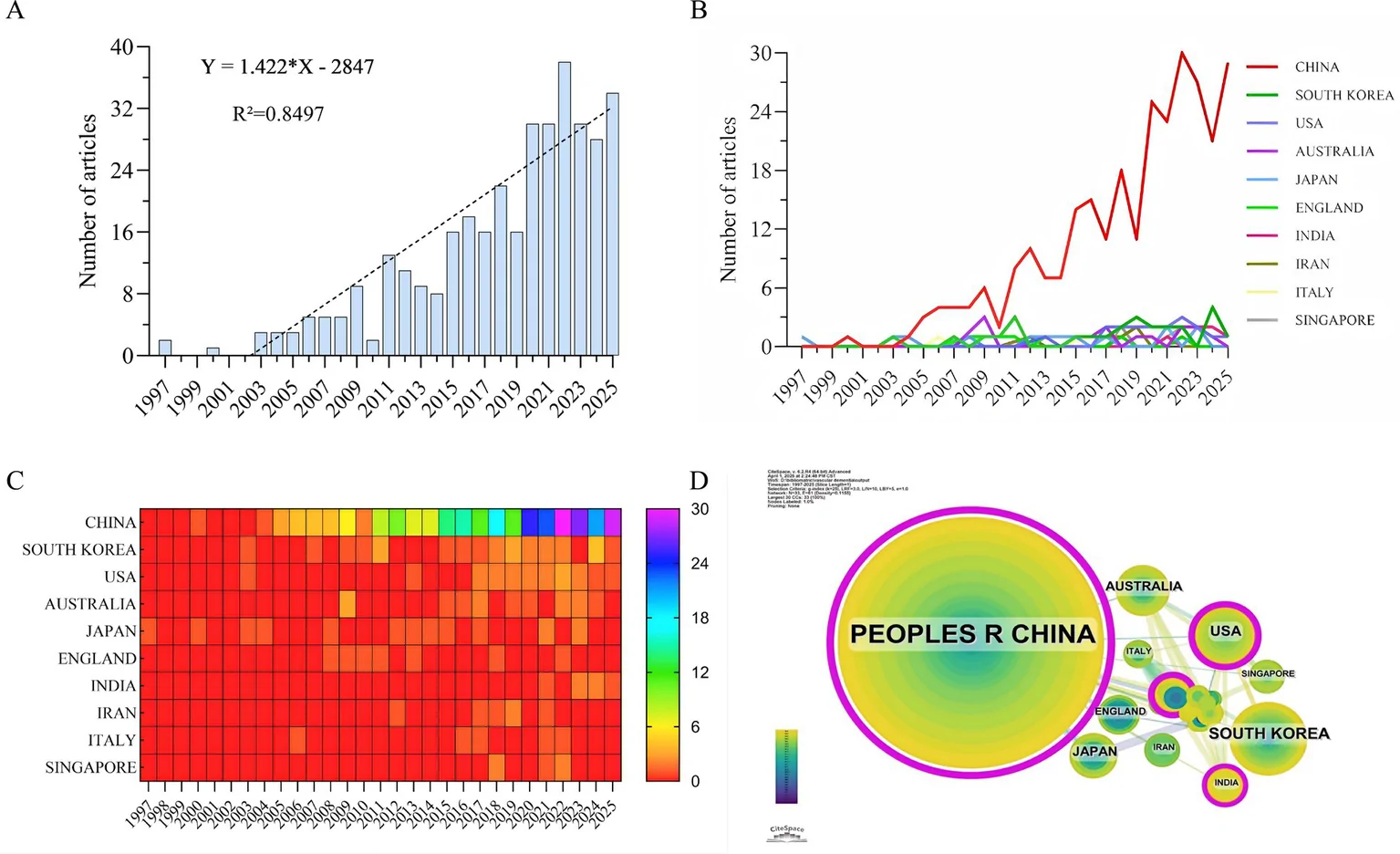
Publication trends and country/region contribution patterns in research on traditional Chinese medicine for vascular dementia. **(A)** Annual number of publications from 1997 to 2025, showing the overall temporal growth of the field. **(B)** Annual publication trends of the top 10 most productive countries/regions from 1997 to 2025, reflecting differences in temporal contribution patterns. **(C)** Global heat map of country/region publication output, illustrating the geographical distribution of research productivity. **(D)** International collaboration network among countries/regions, in which node size represents publication volume and link strength indicates collaboration intensity. Together, this figure summarizes both the temporal development and the global geographical structure of the field.

Overall, the annual number of publications showed a sustained upward trend from 1997 to 2025, indicating increasing scholarly attention to TCM treatment of VaD. Based on changes in the publication curve, the development of this field can be broadly divided into three stages. The period from 1997 to 2008 represented the initial exploratory stage, during which annual publication output remained low and increased slowly, suggesting that the field was still in an early phase of knowledge accumulation. The period from 2009 to 2019 was characterized by rapid growth, with a marked increase in annual publications, indicating that this topic was gradually gaining greater research attention. Since 2020, annual publication output has remained relatively high, suggesting sustained scholarly interest in TCM-related interventions for VaD. However, this bibliometric growth should not be interpreted as direct evidence of methodological maturity, clinical translation, or therapeutic readiness, which require independent evaluation through study-quality assessment, mechanistic validation, and well-designed clinical research.

### 3.2 Country/region contributions and collaboration patterns

Figures 2B–D further illustrate publication trends, spatial distribution, and collaboration patterns at the country/region level in research on TCM treatment of VaD. Figure 2B shows the annual publication trends of the top 10 most productive countries from 1997 to 2025. Figure 2C presents the geographic distribution of publication intensity across countries/regions, whereas Figure 2D depicts the structure of the international collaboration network.

A total of 34 countries/regions contributed to research on TCM treatment of VaD. According to Figures 2B,C and Table 3, the top five most productive countries were China, South Korea, the United States, Australia, and Japan. China ranked first with 281 publications, accounting for 78.71% of the total output, far exceeding all other countries/regions. It was followed by South Korea with 26 publications (7.28%), the United States with 19 publications (5.32%), Australia with 14 publications (3.92%), and Japan with 14 publications (3.92%). China also had the highest total citation count (5,226 citations) and the highest betweenness centrality (0.98), indicating that China was not only the primary contributor to research output in this field but also occupied a key hub position in the international collaboration network. However, this China-centered pattern should be interpreted cautiously. It may be influenced by the topic’s strong association with TCM, the concentration of TCM-specialized institutions in China, domestic research networks, WoSCC database coverage, English-language indexing, and citation practices within this field. Therefore, China’s dominance in publication output reflects both genuine research capacity and structural features of the indexed literature corpus.

**Table 3:** Publication output by country/region.

Rank	Country/region	Article counts	centrality	Percentage (%)	Citation	Citation per publication
1	PRC	281	0.98	78.71%	5,226	18.60
2	South Korea	26	0.01	7.28%	589	22.65
3	USA	19	0.24	5.32%	693	36.47
4	Australia	14	0.07	3.92%	303	21.64
5	Japan	14	0	3.92%	406	29.00
6	England	7	0	1.96%	235	33.57
7	India	6	0.11	1.68%	241	40.17
8	Iran	6	0	1.68%	53	8.83
9	Italy	5	0.09	1.40%	120	24.00
10	Singapore	5	0.04	1.40%	290	58.00

In terms of average citations per publication, China had 18.60 citations per paper, which was lower than Singapore (58.00 citations per paper), India (40.17 citations per paper), the United States (36.47 citations per paper), and the United Kingdom (33.57 citations per paper). This finding suggests that although China has a substantial advantage in publication quantity, the average international influence of individual papers still has room for improvement. It should be noted, however, that average citation count per publication may be affected by multiple factors, including publication year, journal visibility, degree of collaboration, and document type; therefore, this indicator should be interpreted with caution. Similarly, betweenness centrality reflects the structural position of a country or region within the collaboration network, rather than the methodological quality, clinical relevance, or biological validity of the studies produced by that country or region. Therefore, citation counts and centrality values in this study should be understood as bibliometric indicators of visibility and network position, not as direct measures of evidence quality.

Figure 2D shows relatively close collaborative relationships between China and countries such as Italy, Australia, and the United Kingdom. Overall, an international research landscape centered on China has been established in this field. However, beyond a few highly productive countries, the breadth and depth of international collaboration remain insufficient and warrant further strengthening. Importantly, international collaboration itself should not be assumed to automatically improve evidence quality. Its potential value depends on whether it contributes to better study design, standardized diagnostic criteria, transparent reporting, reproducible methods, multicenter clinical trial methodology, and rigorous validation of experimental findings.

### 3.3 Institutional publication output and collaboration characteristics

A total of 475 institutions contributed to research on TCM treatment of VaD. As shown in Table 4, nine of the top 10 most productive institutions were located in China, while Kyung Hee University was located in South Korea. This pattern indicates that research capacity in this field is predominantly concentrated in Chinese universities of traditional Chinese medicine and their affiliated research institutions, although important contributions from institutions outside mainland China should also be recognized. This institutional concentration is expected given the traditional medicine focus of the topic and the long-standing academic, clinical, and experimental infrastructure for TCM research in China. Nevertheless, the pattern may also be shaped by the use of WoSCC as the only database, the English-language restriction, the underrepresentation of Chinese-language journals, and the tendency of domestic TCM research groups to collaborate within established institutional networks. Beijing University of Chinese Medicine ranked first with 36 publications, 890 total citations, and an average of 24.72 citations per publication. Capital Medical University ranked second with 29 publications, but had the highest total citation count among the leading institutions, with 969 citations and an average of 33.41 citations per publication, suggesting a relatively high academic impact. Tianjin University of Traditional Chinese Medicine ranked third, with 27 publications and 656 citations.

**Table 4:** Institutional publication output.

Rank	Institution	Country/region	Number of studies	Total citations	Average citation
1	Beijing University of Chinese Medicine	China	36	890	24.72
2	Capital Medical University	China	29	969	33.41
3	Tianjin University of Traditional Chinese Medicine	China	27	656	24.30
4	China Academy of Chinese Medical Sciences	China	26	344	13.23
5	Xiyuan Hospital	China	19	210	11.05
6	Guangzhou University of Chinese Medicine	China	17	150	8.82
7	Anhui University of Chinese Medicine	China	11	242	22.00
8	Kyung Hee University	South Korea	10	33	3.30
9	Heilongjiang University of Chinese Medicine	China	10	102	10.20
10	China Medical University Taiwan	China	9	165	18.33

Figure 3A shows the institutional collaboration network. Current collaboration among institutions was mainly concentrated among domestic institutions, whereas international institutional collaboration remained limited. These findings suggest that research on TCM treatment of VaD is currently driven primarily by domestic academic networks. In the future, cross-regional, interdisciplinary, and international institutional collaboration should be further strengthened in combination with improvements in study design, diagnostic standardization, intervention reporting, data transparency, reproducibility, and clinical trial methodology, rather than being viewed as a sufficient condition for improving evidence quality by itself.

**Figure 3:**
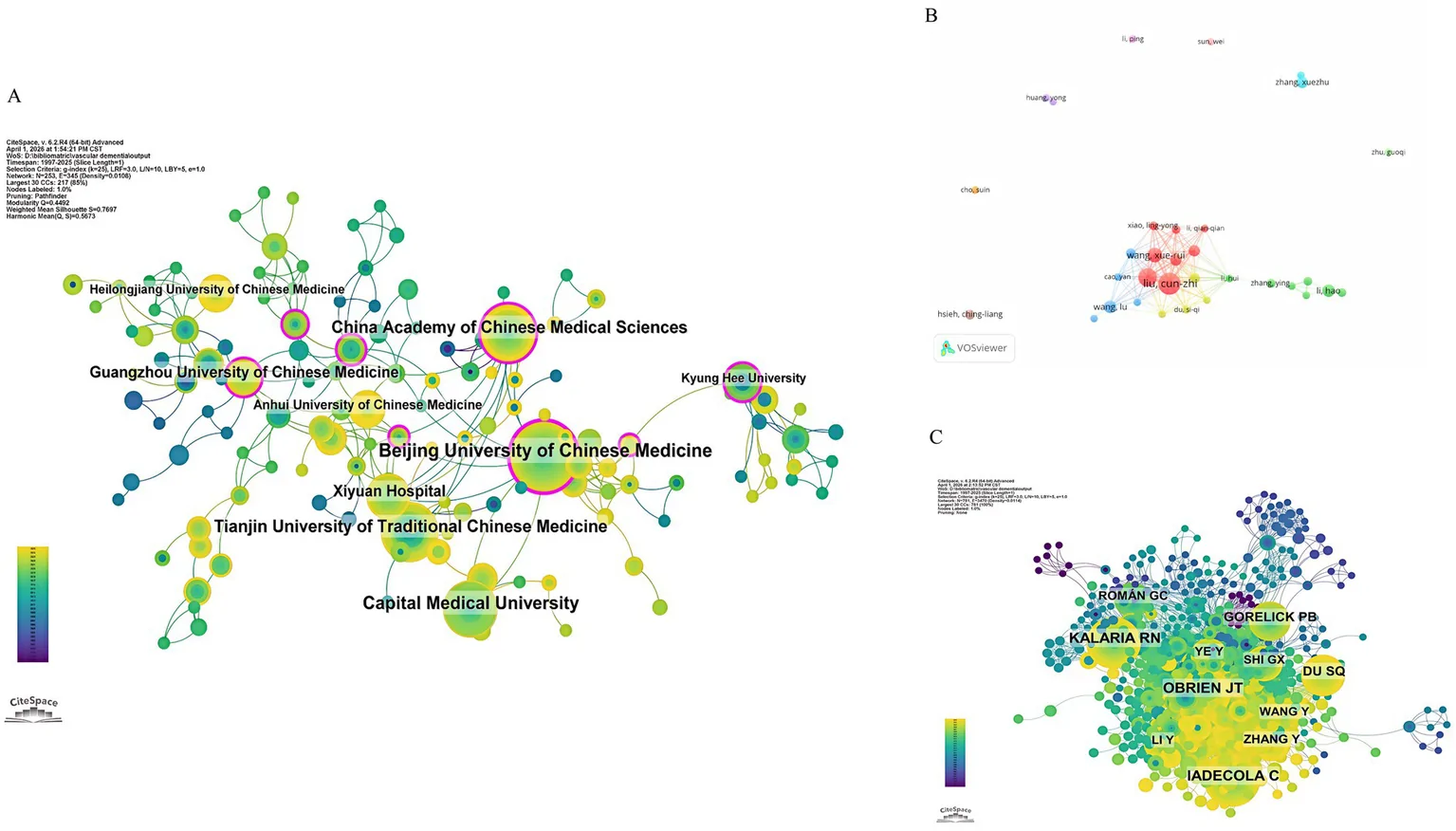
Institution and author collaboration networks in research on Traditional Chinese Medicine for vascular dementia. **(A)** Institutional collaboration network, showing the cooperative relationships among major research institutions. **(B)** Author collaboration network, reflecting cooperation patterns among productive authors. **(C)** Author co-citation network, identifying influential scholars whose work constitutes the intellectual basis of the field. This figure highlights both the production-side collaboration structure and the author-level knowledge influence network.

### 3.4 Journal distribution and characteristics of co-cited journals

Figures 4A–C collectively present the journal landscape and knowledge-source structure of this field from three perspectives: publication journal distribution, co-cited journal structure, and dual-map overlay. Table 1 lists the top 10 journals by publication output in this field. Evidence-Based Complementary and Alternative Medicine ranked first with 22 publications (6.16%), followed by the Journal of Ethnopharmacology with 20 publications (5.60%) and Neural Regeneration Research with 15 publications (4.20%). Most of the leading publication venues were Q1 or Q2 journals in complementary medicine, pharmacology, and neuroscience, indicating that related studies have been disseminated across several established disciplinary journals. Further analysis of the journal publication density map (Figure 4A) showed that high-density areas were mainly concentrated in journals related to complementary and alternative medicine, ethnopharmacology, and neural regeneration. This indicates a clear pattern of journal clustering in publications on TCM treatment of VaD, with research outputs mainly concentrated in a limited number of journals closely related to TCM, natural medicines, and repair of nervous system injury. This distribution suggests that the field has established relatively stable publication venues, while also indicating that its dissemination remains largely concentrated within specific disciplinary communities.

**Figure 4:**
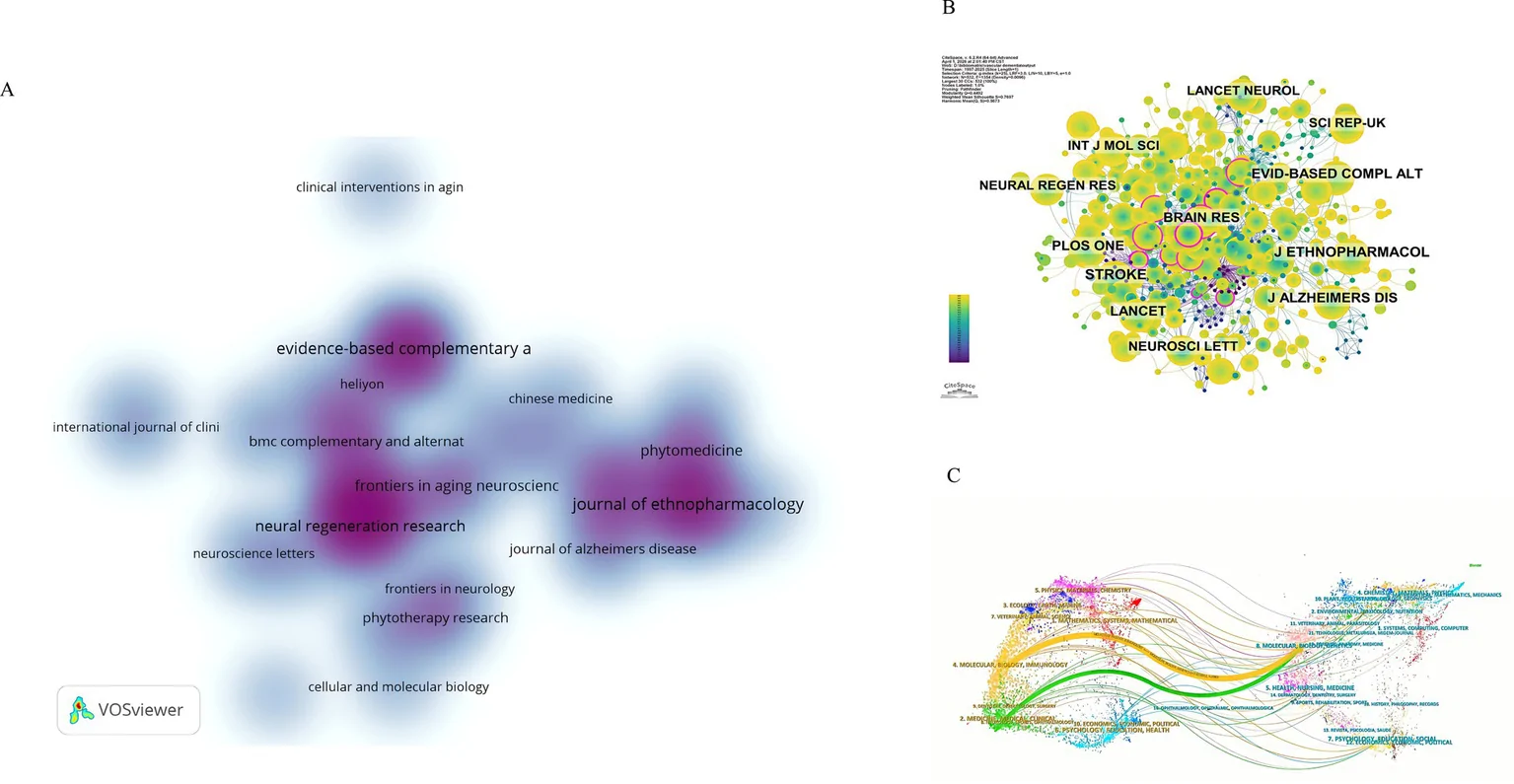
Journal distribution and knowledge-source structure in research on traditional Chinese medicine for vascular dementia. **(A)** Density map of journals publishing related studies, showing the concentration of publications in specific disciplinary outlets. **(B)** Co-cited journal network, identifying highly influential journals constituting the knowledge source base of the field. **(C)** Dual-map overlay of journals, illustrating cross-disciplinary citation trajectories between citing and cited journals. This figure reflects the publication venues, knowledge sources, and interdisciplinary citation patterns of the field.

Co-cited journal analysis was used to identify journals that frequently served as knowledge sources within the retrieved literature corpus. However, co-citation frequency should be interpreted as an indicator of citation attention and intellectual linkage, rather than as direct evidence of methodological quality, clinical importance, or mechanistic validity. As shown in Figure 4B and Table 2, the most frequently co-cited journal was Stroke (169 co-citations), followed by Journal of Ethnopharmacology (141 co-citations) and Evidence-Based Complementary and Alternative Medicine (138 co-citations). Among the top 10 co-cited journals, high-impact general medical journals such as The Lancet were also included, with The Lancet receiving 123 co-citations and having the highest impact factor among these journals (IF = 88.5). This suggests that the knowledge base of TCM treatment for VaD is not confined to TCM or complementary and alternative medicine, but also extensively draws on research from stroke medicine, neuroscience, and clinical medicine. Nevertheless, a journal’s co-citation frequency or impact factor should not be equated with the strength of evidence provided by individual studies cited from that journal. Among the co-cited journals, 80% were ranked in Q1 or Q2. The disciplinary distribution of citing and cited journals was further visualized using a dual-map overlay (Figure 4C). Colored trajectories represent citation links, with citing journals shown on the left and cited journals on the right. The overlay identified two major citation pathways, indicating that studies published in journals in the molecular/biology/genetics fields were mainly cited by journals in the medicine/medical/clinical and molecular/biology/immunology fields. These findings suggest that research on TCM treatment of VaD has shown an increasingly interdisciplinary pattern, extending from basic mechanistic studies toward clinical and translational research.

### 3.5 Core authors and highly influential co-cited authors

Table 5 presents the most productive authors and the authors with the highest co-citation frequencies in this field. Liu Cun-zhi ranked first with 18 publications, followed by Yang Jing-wen with 14 publications and Wang Xue-rui with 10 publications. The sum of the individual publication counts of the 10 most productive authors was 91. Because coauthored publications may be counted for more than one author, this value should not be interpreted as the number or proportion of unique publications contributed by these authors.

**Table 5:** Author publication output and co-citation frequency.

Rank	Author	Count	Rank	Co-cited author	Citation
1	Liu, cun-zhi	18	1	Obrien jt	70
2	Yang, jing-wen	14	2	Iadecola c	65
3	Wang, xue-rui	10	3	Kalaria rn	63
4	Li, hao	8	4	Gorelick pb	42
5	Wang, lu	8	5	Du sq	40
6	Shi, guang-xia	7	6	Román gc	31
7	Zhang, xuezhu	7	7	Ye y	30
8	Zhu, wen	7	8	Zhang y	30
9	Hsieh, ching-liang	6	9	Shi gx	29
10	Ma, si-ming	6	10	Wang y	28

As shown in the author collaboration network (Figure 3B), certain collaborative relationships have formed among core authors, although the overall network remains relatively dispersed.

Figure 3C and Table 5 show the top 10 co-cited authors and their citation frequencies. Eight authors were co-cited at least 30 times, indicating that their work has substantial academic recognition and influence. The largest nodes corresponded to the most frequently co-cited authors, including O’Brien JT (70 co-citations), Iadecola C (65 co-citations), and Kalaria RN (63 co-citations), who were the most frequently co-cited authors in the retrieved literature. These scholars mainly focused on the definition, diagnosis, epidemiology, and pathophysiological mechanisms of VaD, suggesting that the theoretical foundation of TCM research on VaD is closely connected with the international mainstream research framework of VaD.

### 3.6 Co-cited reference analysis and evolution of the knowledge base

Figures 5A–C illustrate the formation and evolution of the knowledge base in research on TCM treatment of VaD from three perspectives: the co-cited reference network, clustering structure, and temporal evolution.

**Figure 5:**
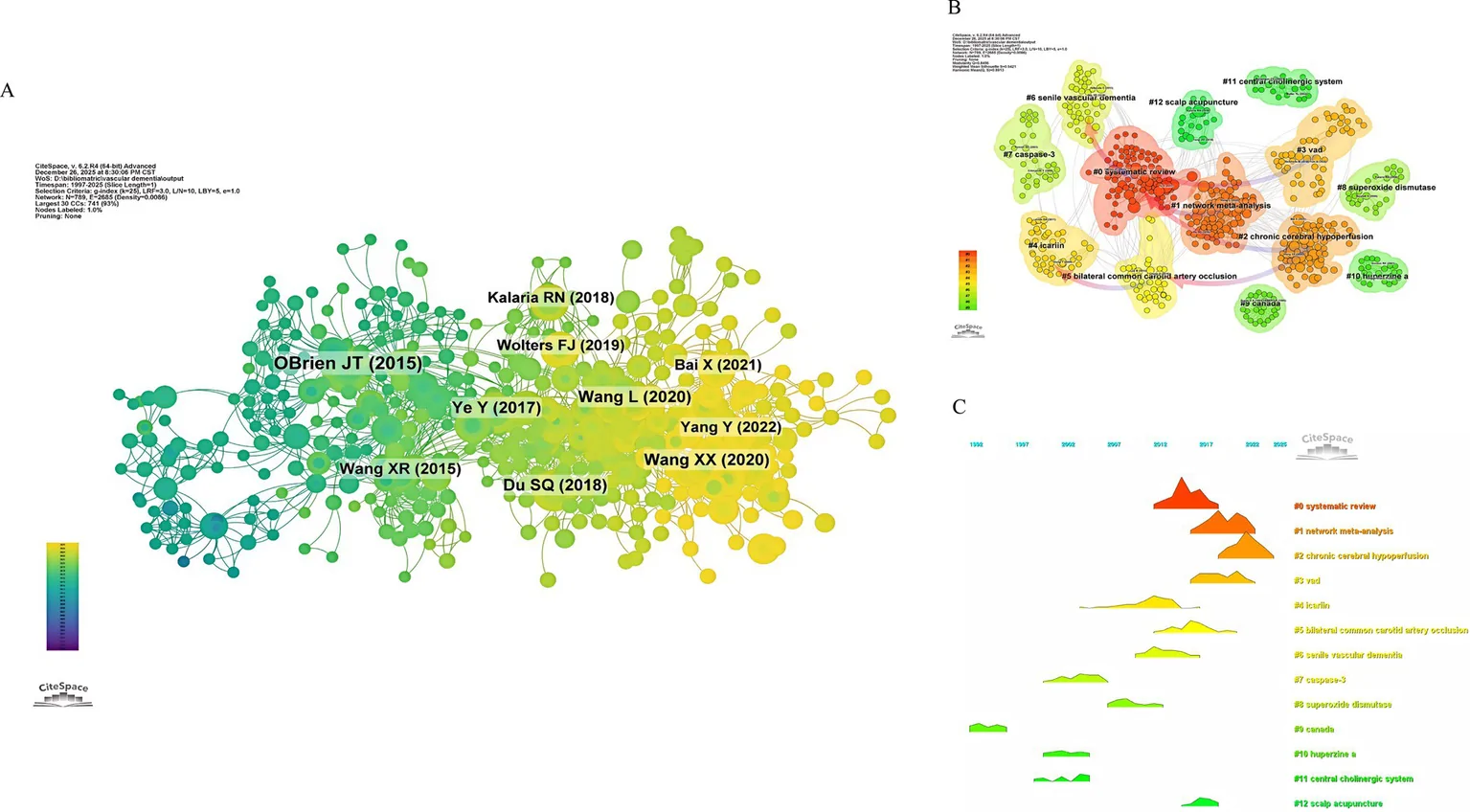
Co-cited reference network and evolution of the knowledge base in research on traditional Chinese medicine for vascular dementia. **(A)** Co-cited reference network, presenting the overall structure of highly co-cited literature. **(B)** Reference clustering map, showing the major thematic clusters of the knowledge base. **(C)** Reference timeline/ridge view, illustrating the temporal evolution of key co-cited themes and research emphases. This figure demonstrates how the intellectual foundation of the field has evolved from early mechanistic studies to more structured and diversified thematic development.

Using 1 year as the time slice and covering the period from 1997 to 2025, the co-cited reference network contained 1,004 nodes and 2,877 links (Figure 5A), indicating that a relatively rich and interconnected knowledge structure has been established in this field. Co-cited reference analysis identifies publications that are frequently cited together and therefore helps reveal the intellectual structure of the field. However, high co-citation frequency does not necessarily indicate superior methodological rigor, clinical importance, or validated mechanistic conclusions. These references should be interpreted as influential or frequently used knowledge sources within the bibliometric network, rather than as definitive evidence of efficacy or mechanism. The top co-cited references indicate that the intellectual foundation of TCM-related research on VaD is mainly organized around four interconnected knowledge domains. First, several highly co-cited publications provide the disease-level framework for VaD, including its definition, nosology, diagnostic uncertainty, epidemiological burden, and relationship with broader vascular cognitive impairment and mixed dementia. These references form the conceptual basis on which TCM-related studies define the target disease and interpret cognitive outcomes. Second, a group of references focuses on experimental models and pathological mechanisms, especially chronic cerebral hypoperfusion, bilateral common carotid artery occlusion, oxidative stress, inflammation, apoptosis, autophagy, microglial activation, and hippocampal injury. These studies provide the dominant biological framework for explaining VaD-related cognitive impairment in preclinical research. However, the prominence of these references also indicates that the current bibliometric landscape may be strongly shaped by a limited set of preclinical models, particularly chronic cerebral hypoperfusion and bilateral common carotid artery occlusion models. Although these models are useful for studying hypoperfusion-induced neuronal injury, oxidative stress, neuroinflammation, and hippocampal dysfunction, they cannot fully reproduce the clinical heterogeneity of human VaD/VCI, including ageing, hypertension, diabetes, cerebral small vessel disease, white matter lesions, strategic infarcts, post-stroke cognitive impairment, and mixed Alzheimer-related pathology. Therefore, the mechanistic emphasis revealed by co-citation analysis should be interpreted as model-influenced research attention rather than as a complete representation of human VaD pathobiology. Third, acupuncture-related references occupy an important position in the co-citation network, reflecting sustained attention to acupuncture, electroacupuncture, scalp acupuncture, hippocampal plasticity, neuroinflammatory regulation, and oxidative stress-related mechanisms. Fourth, TCM intervention-oriented reviews and mechanistic studies connect traditional therapeutic modalities with modern neurobiological concepts, thereby linking herbal medicine, natural products, and acupuncture research with the broader VaD knowledge base.

Therefore, the co-cited references should not be interpreted merely as a list of influential individual papers. Rather, they collectively suggest that the field has been shaped by the convergence of VaD diagnostic frameworks, animal model-based mechanistic research, acupuncture-related neuroprotective studies, and TCM intervention research. This knowledge structure also explains why subsequent keyword clusters and burst terms are concentrated around chronic cerebral hypoperfusion, oxidative stress, inflammation, apoptosis, hippocampus, acupuncture, network pharmacology, and evidence synthesis. The co-citation clustering map and timeline view (Figures 5B,C) showed that early research hotspots were mainly concentrated in topics related to neurotransmitter regulation and apoptosis, such as the cholinergic system (cluster 11), Huperzine A (cluster 10), and caspase-3 (cluster 7). During the middle stage, research attention gradually shifted toward themes such as systematic review (cluster 0), icariin (cluster 3), senile vascular dementia (cluster 6), and superoxide dismutase (cluster 8). In recent years, hotspots have further evolved toward network meta-analysis (cluster 1), chronic cerebral hypoperfusion (cluster 2), VaD (cluster 3), bilateral common carotid artery occlusion (cluster 5), and scalp acupuncture (cluster 12). Overall, research on TCM treatment of VaD has gradually transitioned from early exploration centered on single compounds, single targets, and basic injury mechanisms to greater bibliometric attention to evidence integration, disease model standardization, and refined evaluation of specific interventions. This trend reflects changes in research topics and citation structures, rather than conclusive evidence that the field has achieved methodological maturity or clinical readiness (Table 6).

**Table 6:** Co-cited references.

Rank	Title	Journal	Author	Co-citations
1	Vascular dementia	The Lancet	OBrien JT	21
2	Acupuncture inhibits TXNIP-associated oxidative stress and inflammation to attenuate cognitive impairment in vascular dementia rats	CNS Neuroscience and Therapeutics	Du SQ	15
3	Inflammation, apoptosis and autophagy as critical players in vascular dementia	European Review for Medical and Pharmacological Sciences	Wang XX	15
4	Acupuncture Attenuates Inflammation in Microglia of Vascular Dementia Rats by Inhibiting miR-93-Mediated TLR4/MyD88/NF-κB Signaling Pathway	Oxidative Medicine and Cellular Longevity	Wang L	14
5	Acupuncture Attenuated Vascular Dementia-Induced Hippocampal Long-Term Potentiation Impairments via Activation of D1/D5 Receptors	Stroke	Ye Y	14
6	The pathology and pathophysiology of vascular dementia	Neuropharmacology	Kalaria RN	13
7	Vascular dementia: A microglia?s perspective	Ageing Research Reviews	Yang Y	12
8	Traditional Chinese Medicine Intervenes in Vascular Dementia: Traditional Medicine Brings New Expectations	Frontiers in Pharmacology	Bai X	11
9	Epidemiology of Vascular Dementia Nosology in a Time of Epiomics	Arteriosclerosis, Thrombosis, and Vascular Biology	Wolters FJ	11
10	Mechanisms of acupuncture on vascular dementia-A review of animal studies	Neurochemistry International	Ye Y	11

### 3.7 Keyword co-occurrence analysis, thematic clustering, and frontier evolution

Figures 6–8 collectively illustrate the formation of research hotspots and the evolution of frontiers in TCM treatment of VaD from three perspectives: high-frequency keyword distribution, clustering structure, and burst analysis. Specifically, Figures 6A,B show the keyword co-occurrence network and density distribution generated using VOSviewer, whereas Figures 7A,B present the keyword clustering structure and temporal evolution generated using CiteSpace and Figures 8A,B display the burst characteristics of co-cited references and keywords.

**Figure 6:**
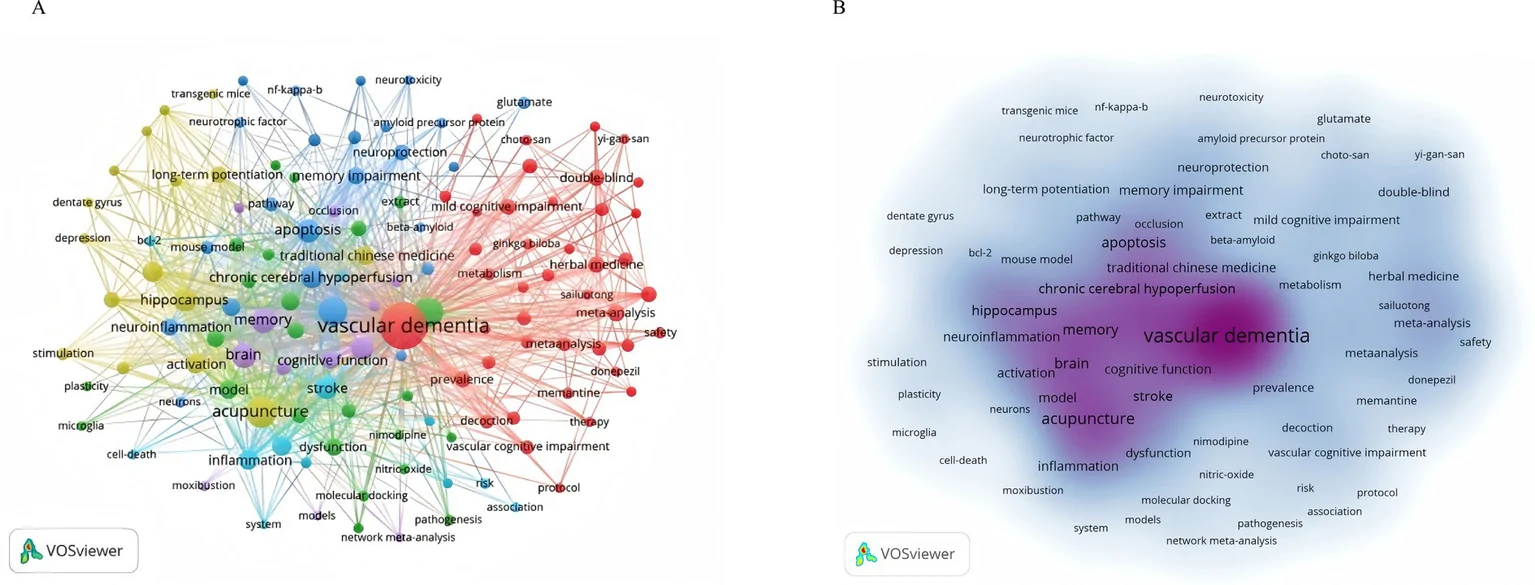
High-frequency keyword co-occurrence network and density distribution in research on Traditional Chinese Medicine for vascular dementia. **(A)** Keyword co-occurrence network showing the relationships among major high-frequency terms. **(B)** Keyword density map highlighting the concentration and relative importance of research hotspots. This figure provides an overview of the central research topics and their interrelationships in the field.

**Figure 7:**
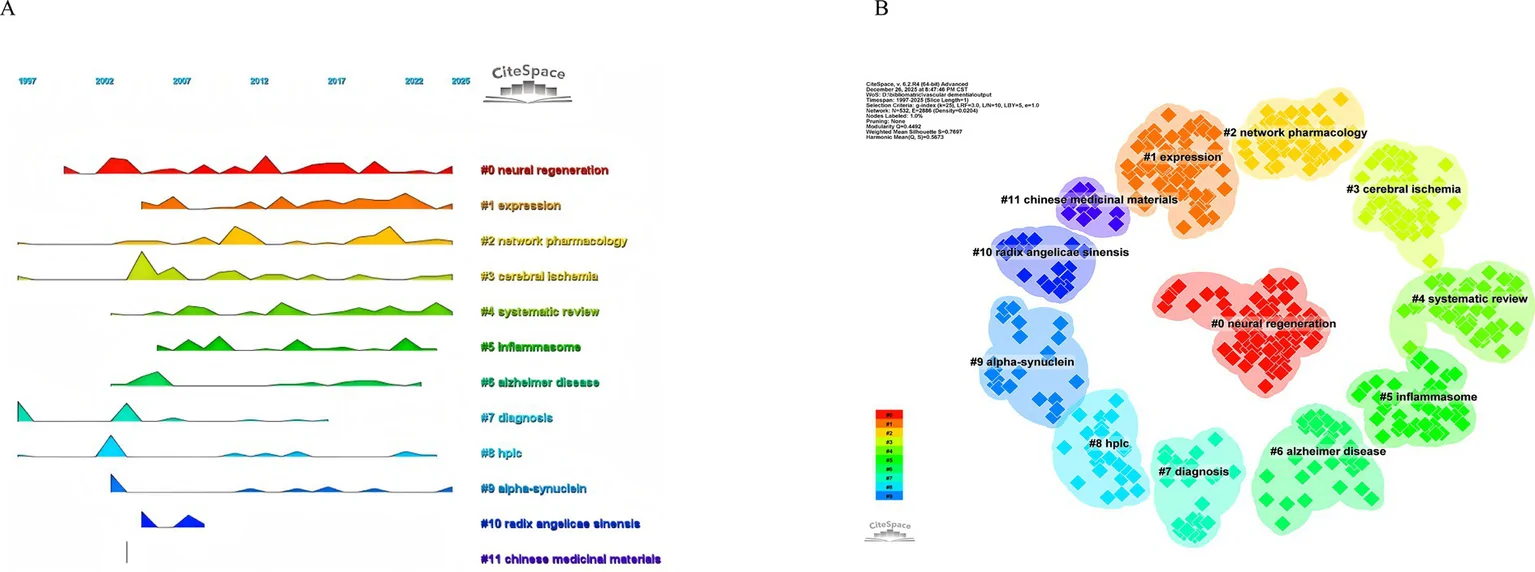
Keyword clustering structure and thematic evolution in research on traditional Chinese medicine for vascular dementia. **(A)** Keyword clustering ridge/timeline view, showing the temporal distribution and evolutionary trajectory of major thematic clusters. **(B)** Keyword clustering map, presenting the structural organization of major research themes. This figure reveals the thematic stratification of the field and the transition from traditional efficacy-oriented studies to mechanism- and technology-oriented research.

**Figure 8:**
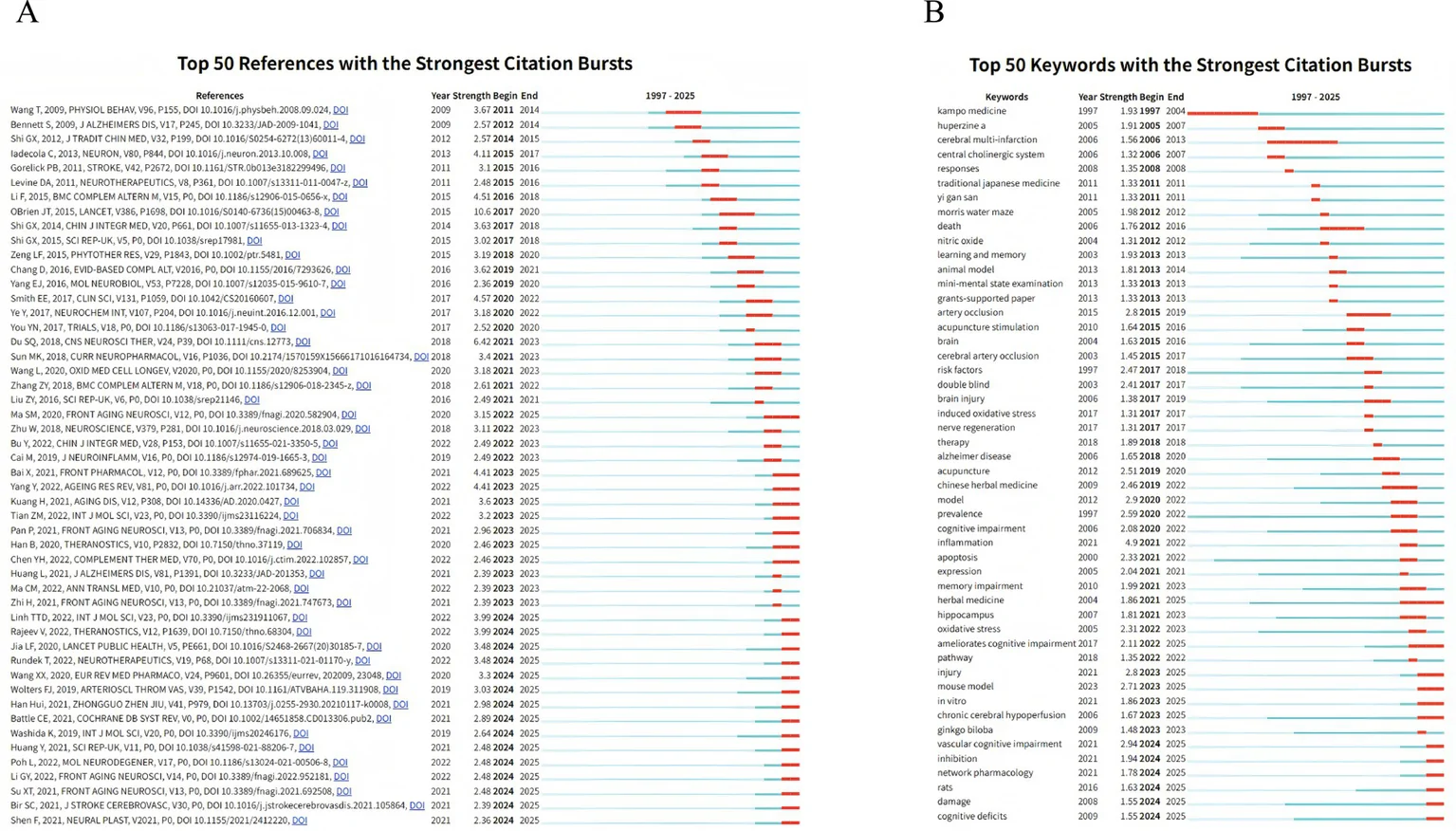
Burst analysis of co-cited references and keywords in research on traditional Chinese medicine for vascular dementia: **(A)** Burst detection of co-cited references, identifying influential references that experienced strong citation increases over time. **(B)** Burst detection of keywords, showing research topics that gained rapid attention in different periods. This figure captures both the changing intellectual basis and the evolving research frontiers of the field.

Keyword analysis provides an intuitive reflection of research hotspots and thematic evolution. After keyword harmonization synonymous terms and abbreviations were merged while broad high-frequency terms were retained only when they met the predefined threshold and reflected recurrent indexing patterns in the original dataset. Therefore general terms such as “brain,” “impairment,” “mechanisms,” and “model” were reported descriptively in the high-frequency keyword table but were not overinterpreted as specific research themes. As shown in Table 7 and Figures 6A,B the most frequently occurring keywords were “vascular dementia” (225 occurrences) “acupuncture” (83 occurrences) “cognitive impairment” (72 occurrences) “oxidative stress” (66 occurrences) and “brain” (47 occurrences). The high frequency of “acupuncture” indicates that acupuncture-related research represents a major independent branch of this field rather than merely a subsidiary component of herbal or plant-derived intervention research. Therefore the current TCM-VaD literature should be interpreted as a heterogeneous research field composed of at least two major branches: Herbal/natural product-based pharmacological studies and acupuncture/moxibustion-based non-pharmacological intervention studies. In addition keywords such as “memory,” “apoptosis,” “hippocampus,” “stroke,” and “chronic cerebral hypoperfusion” also appeared frequently indicating that current research primarily focuses on the improvement of cognitive impairment cerebral hypoperfusion-induced injury and related neurobiological mechanisms. Within the former branch, natural products and plant-derived active compounds represent the component most directly related to component characterization and plant-based neuroprotective research.

**Table 7:** High-frequency keywords.

Rank	Keyword	Counts	Rank	Keyword	Counts
1	Vascular dementia	225	11	impairment	36
2	Acupuncture	83	12	electroacupuncture	28
3	Cognitive impairment	72	13	inflammation	26
4	Oxidative stress	66	14	activation	25
5	Brain	47	15	mechanisms	25
6	Memory	45	16	traditional chinese medicine	24
7	Apoptosis	41	17	model	22
8	Hippocampus	39	18	cerebral-ischemia	21
9	Stroke	37	19	cognitive function	21
10	Chronic cerebral hypoperfusion	36	20	rat model	20

Keywords were manually standardized before analysis. Abbreviations and spelling variants were harmonized, such as “VaD/VD” to “vascular dementia,” and “TCM” to “traditional Chinese medicine.” Broad terms that met the frequency threshold, including “brain,” “impairment,” “mechanisms,” and “model,” were retained for transparency but were interpreted cautiously and not treated as independent mechanistic themes.

In the VOSviewer analysis, after removing nonspecific keywords, a co-occurrence network comprising 130 high-frequency keywords was constructed, forming six major clusters. Because several biological processes, such as oxidative stress, inflammation, apoptosis, autophagy, and cell death, are mechanistically interconnected in VaD pathobiology, these clusters should not be interpreted as completely independent mechanistic domains. Instead, they represent partially overlapping thematic modules that reflect different levels of the VaD pathological cascade and different TCM-related intervention approaches. The six clusters can be refined as follows:

Cluster 1: clinical evaluation and herbal intervention context. This cluster included terms such as “vascular dementia,” “decoction,” “diagnosis,” “efficacy,” “herbal medicine,” “protocol,” “safety,” “therapy,” “trial,” and “ginseng.” This module mainly reflects clinical and translational research related to herbal formulas, treatment evaluation, safety assessment, and trial design. However, the presence of terms such as “efficacy,” “trial,” “protocol,” and “safety” should be interpreted as indicating clinical research attention rather than as evidence of clinical effectiveness or high-quality trial design. The clinical value of these studies still depends on whether VaD diagnostic criteria are clearly defined, whether cognitive and functional outcomes are standardized, whether intervention protocols are reproducible, whether appropriate comparators and blinding methods are used, and whether adverse events and follow-up outcomes are adequately reported. Biologically, it is less focused on a single pathway and more closely related to how TCM interventions are evaluated in VaD populations or VaD-related experimental settings.

Cluster 2: experimental injury models and neurovascular dysfunction. This cluster included terms such as “cognitive impairment,” “angiogenesis,” “injury,” “dysfunction,” “model,” “microglia,” “molecular docking,” “nitric oxide,” and “nimodipine.” This module is more closely linked to experimental modeling of VaD-related injury, especially neurovascular dysfunction, microglial activation, endothelial/vascular regulatory responses, and candidate pharmacological mechanisms. These terms suggest that part of the field attempts to connect TCM-related interventions with vascular injury, inflammatory cell responses, and impaired neurovascular regulation. Nevertheless, because many preclinical studies rely on relatively simplified injury models, this cluster may overrepresent acute or chronic hypoperfusion-related mechanisms while underrepresenting ageing-related vascular degeneration, metabolic comorbidities, diffuse small-vessel pathology, and mixed vascular–neurodegenerative lesions that are common in clinical VaD.

Cluster 3: This cluster included terms such as “oxidative stress,” “apoptosis,” “rat model,” “autophagy,” “cognitive deficits,” “neurons,” “memory impairment,” “NF-κB,” “pathway,” “neurotoxicity,” and “receptor.” Rather than representing separate mechanisms, oxidative stress, apoptosis, autophagy, and NF-κB-related signaling should be viewed as an interconnected injury-response axis downstream of chronic cerebral hypoperfusion and ischemic stress. This module corresponds to one of the dominant preclinical frameworks in VaD research, in which sustained hypoperfusion induces oxidative damage, inflammatory signaling, neuronal injury, and cognitive decline. However, this model-centered framework may preferentially highlight neuronal and hippocampal injury pathways and may not sufficiently capture white matter damage, blood–brain barrier dysfunction, microvascular rarefaction, strategic infarcts, or mixed Alzheimer-type protein pathology.

Cluster 4: acupuncture-related hippocampal plasticity and neuromodulation. This cluster included terms such as “activation,” “acupuncture,” “dentate gyrus,” “hippocampus,” “stimulation,” “neural regeneration,” and “transgenic mice.” This module reflects the distinctive position of acupuncture and electroacupuncture in VaD research. Its biological emphasis is not limited to anti-inflammatory or antioxidant effects, but extends to hippocampal plasticity, dentate gyrus-associated neurogenesis, neuromodulation, and functional recovery of learning and memory-related brain regions.

Cluster 5: cerebral blood flow, chronic hypoperfusion, and moxibustion-related vascular regulation. This cluster included terms such as “blood flow,” “brain,” “damage,” “hypoperfusion,” “memory,” “moxibustion,” and “occlusion.” This module is closely related to the upstream vascular component of VaD pathobiology, particularly chronic cerebral hypoperfusion, cerebral blood-flow disturbance, vascular occlusion models, and memory impairment. It also suggests that moxibustion-related studies may be more often interpreted through vascular regulation, thermal/somatosensory stimulation, and improvement of perfusion-related injury. At the same time, the frequent appearance of “hypoperfusion” and “occlusion” indicates that the field’s mechanistic map is partly driven by model availability and experimental operability. Therefore, findings derived from occlusion- or hypoperfusion-based animal models should be translated cautiously to the diverse clinical spectrum of human VaD.

Cluster 6: stroke-related inflammation, cell death, and mixed vascular-neurodegenerative injury. This cluster included terms such as “artery occlusion,” “Bcl-2,” “cell death,” “inflammation,” “risk,” “stroke,” and “system.” This module connects vascular events with downstream inflammatory and cell-death responses. It may also reflect the overlap between VaD, post-stroke cognitive impairment, and mixed vascular-neurodegenerative mechanisms, although the present dataset remains VaD-centered and should not be generalized to the entire VCI/VCID field.

Taken together, the six clusters can be better understood as a pathobiological continuum rather than six isolated topics. Chronic cerebral hypoperfusion, vascular occlusion, and impaired cerebral blood flow represent major upstream vascular insults; these changes may contribute to neurovascular unit disruption, endothelial dysfunction, blood–brain barrier injury, white matter vulnerability, and hippocampal damage. Downstream responses include oxidative stress, microglial activation, neuroinflammation, apoptosis, autophagy imbalance, and neuronal injury, ultimately contributing to memory impairment and cognitive decline. Within this framework, herbal formulas, single herbs, natural products, and active compounds are mainly discussed in relation to multi-target regulation of inflammatory, oxidative, apoptotic, autophagic, and vascular pathways, whereas acupuncture, electroacupuncture, and moxibustion are more closely associated with neuromodulation, hippocampal plasticity, cerebral perfusion, and functional recovery. Therefore, the keyword clusters reveal not only topical hotspots but also how TCM-related interventions are being mapped onto the vascular–neural injury cascade of VaD.

In the burst analysis of cited references and keywords, 50 references and 50 keywords with the strongest citation bursts are presented in Figure 8. Among the burst references shown in Figure 8A, the classical studies with higher citation strength mainly focused on the definition, epidemiology, pathophysiological mechanisms, and therapeutic challenges of VaD. This indicates that research on TCM treatment of VaD has consistently been grounded in a deepening understanding of VaD as a disease entity. However, citation burst strength reflects a rapid increase in citation attention during a specific period and should not be interpreted as direct evidence of methodological quality, clinical significance, or mechanistic validity. Burst references and burst keywords are therefore used in this study to identify evolving research attention and emerging topics, rather than to determine the evidentiary strength of specific interventions or mechanisms. The 50 burst references were published between 1997 and 2025 and have been frequently cited over the past three decades. Notably, 23 of these references are currently in an active citation burst period.

Meanwhile, examination of the 50 keywords with the strongest bursts revealed a thematic transition (Figure 8B) revealed a clear thematic transition. Burst keywords have gradually shifted from broad descriptions of diseases and therapeutic approaches in the early stage to more mechanism-oriented and technology-integrated topics, such as “network pharmacology,” “inflammasome,” “HPLC,” and “alpha-synuclein.” This pattern suggests increasing bibliometric attention to component characterization, hypothesis-generating network analysis, neuroinflammatory mechanisms, and mixed vascular–neurodegenerative themes. However, the emergence of these terms should not be interpreted as proof that the field has achieved validated mechanistic maturation. In particular, network pharmacology is often used as a predictive and hypothesis-generating approach, and its mechanistic implications require further confirmation through chemical characterization, quality control, target validation, dose–response experiments, pathway-specific intervention, and independent experimental or clinical verification. Therefore, the burst analysis reflects methodological popularity and emerging research directions, rather than established causal mechanisms or confirmed therapeutic targets.

## 4 Discussion

This study conducted a systematic bibliometric analysis of publication trends, country and institutional distribution, author collaboration, journal characteristics, co-cited references, and keyword evolution in the field of TCM treatment for VaD from 1997 to 2025, based on the WoSCC database. Overall, research on TCM treatment of VaD has gradually progressed from early exploration to sustained development, showing an evolutionary pattern characterized by concentrated research capacity, deepening mechanistic investigation, diversification of intervention modalities, and increasing integration of frontier technologies.

### 4.1 Developmental trends of the field

The publication trend showed an overall year-by-year increase in research on TCM treatment of VaD, with particularly marked growth after 2009. This change indicates that TCM intervention for VaD has gradually moved from early, scattered exploration toward a more established stage. During this period, VaD/VCI-related research progressively expanded from the concept of a “dementia subtype” to the broader continuum of vascular cognitive impairment. The increasing clarification of research objects, diagnostic boundaries, and disease spectrum has provided a more stable conceptual framework for subsequent clinical and basic studies (1).

In 2011, the American Heart Association/American Stroke Association issued a scientific statement on “vascular contributions to cognitive impairment and dementia,” systematically emphasizing the importance of vascular factors in cognitive impairment and promoting a shift in the field from disease nomenclature alone toward a research perspective that integrates mechanisms and prevention (2). Subsequently, efforts such as those by VASCOG further advanced the standardization of diagnostic frameworks for VCI/VaD (34). Since then, academic attention to this field has continued to increase.

On the one hand, VaD/VCI is not a single disease entity but a clinical syndrome with pronounced heterogeneity and continuum characteristics. It involves multiple pathological substrates, including cerebral small vessel disease, post-stroke injury, hypoperfusion, and mixed pathology. At present, clinical management still relies mainly on risk-factor control and prevention, and specific therapeutic strategies supported by robust evidence remain lacking (5, 8). As an important type of cognitive impairment, VaD involves multiple pathological processes, including impaired cerebral blood flow, neuroinflammation, oxidative stress, synaptic injury, and neuronal apoptosis. TCM is characterized by multi-component, multi-target, and holistic regulatory effects, which has been proposed as a rationale for investigation with the multi-pathway and multilevel pathological processes of this disease. This provides both a disciplinary foundation and a real-world demand for the continued growth of related research (25).

From the developmental trajectory of TCM treatment for VaD, animal models and classical mechanistic studies involving oxidative stress, apoptosis, and inflammation gradually matured from the late 2000s to around 2010 (35). This maturation helped standardize the experimental framework for interventions such as Chinese herbal formulas, natural products, acupuncture, and moxibustion, making them more compatible with scientific research paradigms (36, 37). Therefore, the growth in publication output during this period does not merely indicate that “researchers began to pay attention” to the topic; rather, it suggests that the field had begun to develop relatively stable scientific awareness, research models, and technical pathways. This interpretation is also consistent with the keyword clusters containing terms such as “oxidative stress,” “apoptosis,” “model,” “microglia,” and “nitric oxide,” indicating that early growth was largely driven by the expansion of disease models and classical injury-mechanism studies (38).

### 4.2 Country and institutional influence patterns

The results showed that China exhibited a clear pattern of “high output, high connectivity, and high concentration” in this field. This pattern should not be simply attributed to the geographic nature of TCM research; rather, it should be understood as the result of three interacting factors: the inherent advantages of traditional medicine, the background of disease burden, and the compatibility between research pathways and the characteristics of VaD (7, 39, 40). Research on TCM treatment of VaD relies not only on TCM theory and medicinal resources but also on support from neurology, cerebrovascular medicine, pharmacology, and experimental medicine platforms. China has long-standing foundations in TCM university systems, affiliated TCM hospitals, accumulated experience with classical formulas, and animal experimental platforms. These advantages make it easier to form continuous research teams and stable mechanisms for generating research questions, thereby establishing a relatively complete knowledge-production chain (41).

Furthermore, China’s prominent centrality suggests that its role is not limited to being the major source of publication output; it also serves as a key organizer and connecting node in the collaboration network of this field. On the one hand, many research topics, intervention objects, and experimental approaches have been continuously advanced by Chinese research teams. On the other hand, international collaboration, citation, and knowledge dissemination related to this field are also frequently connected through China as a central node (42).

However, China’s average citation count per publication was lower than that of several countries with smaller publication outputs. At a deeper level, this phenomenon may reflect a structural feature within the field: the expansion of publication scale has outpaced the accumulation of landmark studies. A large proportion of Chinese publications remain focused on local mechanistic validation, single-model studies, or regionally oriented research topics, whereas truly representative studies that can enter the mainstream international cognitive research framework and be widely cited across disciplines remain relatively limited (21, 39).

From this perspective, China’s current strengths and limitations have the same underlying source. Its strong domestic research foundation and large research workforce have generated a clear quantitative advantage; however, the dispersion of research topics, strong internal circulation, insufficient internationally accessible expression, and limited high-level integration have prevented this quantitative advantage from being fully translated into a corresponding average citation impact (40). Therefore, the key to further enhancing China’s academic influence in this field lies in developing research questions with stronger international explanatory power. Specifically, future studies should strengthen multicenter, high-quality, and reproducible clinical and basic research while reducing low-level repetition. At the same time, greater attention should be paid to mechanistic integration, standardized diagnostic frameworks, validation of key targets, and international academic communication, so that research findings can be more effectively incorporated into the mainstream narrative of international VaD/VCI research (43).

### 4.3 Core research groups and authors

The author collaboration network showed that a collaborative group centered on Professor Liu Cun-zhi’s team has formed in this field, highlighting the team’s central role in research on vascular cognitive impairment and dementia. Based on published studies, Liu’s team has pursued a parallel trajectory of clinical and basic research in this area. Their work has progressed from clinical efficacy evaluation to neurobiological mechanism exploration, with a primary focus on mechanisms such as oxidative stress (44), mitochondrial function (45), synaptic plasticity (46), and neuroinflammation (47). Through the integration of multi-omics research (48) and subsequent clinical validation, the team has advanced mechanistic evidence toward brain imaging and clinically stratified benefits. This reflects an overall trend from parallel clinical and basic research toward translational and integrative research. The team has provided important support for methodological innovation and theoretical deepening in multiple areas related to TCM-based improvement of cognitive impairment, and its research approaches and conceptual framework are worthy of reference.

It is also noteworthy that, based on currently available publications, the Hsieh team in Taiwan appears to have followed a relatively independent developmental trajectory, with limited collaboration with mainland scholars represented by Liu’s team. The Taiwan-based team has mainly focused on neuroregeneration and brain functional network regulation, with an emphasis on cross-disease mechanistic research (49–51). Overall, this trajectory has developed in parallel with mainland research teams, and no evidence of co-authored collaboration has yet been identified. Nevertheless, the two lines of research may have strong potential complementarity in areas such as post-stroke cognitive impairment, brain network remodeling, and multicenter clinical studies.

However, the collaboration network indicates that current cooperation remains mainly concentrated among domestic institutions, while international collaboration and interdisciplinary integration are still relatively limited. VaD research is inherently interdisciplinary, involving neurology, geriatrics, cerebrovascular medicine, pharmacology, rehabilitation medicine, and evidence-based medicine. If collaboration networks remain confined to relatively closed academic circles over the long term, this may limit research perspectives, the level of evidence, and international recognition. Therefore, future research should promote deeper collaboration among basic researchers, clinicians, and methodologists, while strengthening exchanges with international teams in cerebrovascular disease and dementia research, thereby improving standardization and international comparability.

### 4.4 Framework of the knowledge system

The keyword clustering and burst analyses indicated that the TCM–VaD literature represented in the present dataset has developed a relatively clear thematic hierarchy (52). The knowledge structure of TCM treatment for VaD is no longer limited to the early stage of scattered disease descriptions and therapeutic observations; instead, it has gradually formed a clearer thematic stratification and evolutionary trajectory. Based on high-frequency and frontier keywords, this field continues to focus on classical pathological processes such as cognitive impairment, chronic cerebral hypoperfusion, oxidative stress, apoptosis, and hippocampal injury (53). At the same time, it is extending toward topics with greater mechanistic depth and technological integration, including inflammasomes, neuroregeneration, expression profile changes, component characterization, and network pharmacology (54). These findings suggest that research on TCM treatment of VaD is undergoing a transition from asking “whether it is effective” to exploring “how it works and which key pathological links it targets.” The research focus has clearly shifted from simple efficacy observation to mechanistic interpretation and elucidation of therapeutic networks.

Furthermore, the emergence of burst keywords such as “network pharmacology,” “HPLC,” “inflammasome,” and “alpha-synuclein” does not merely indicate methodological updates; rather, it suggests that the field is attempting to address two long-standing issues in TCM research: the unclear material basis and insufficient mechanistic explanation. The former has encouraged researchers to place greater emphasis on the identification of active components, clarification of the material basis of formulas, and quality control (55, 56). The latter has promoted a shift from single-pathway validation toward mechanistic integration involving multiple targets, multiple levels, and multiple technologies. These frontier keywords indicate that the field is transitioning from experience-based research toward a more mechanistic, standardized, and interpretable research paradigm. This evolution also suggests that the accumulation of primary research evidence in this field has reached a certain scale. Researchers are no longer satisfied with repeatedly validating single models and single indicators; instead, systematic reviews, evidence synthesis, and methodological reflection are increasingly needed to identify robust conclusions and translatable therapeutic targets. Therefore, the field has begun to move from primary evidence accumulation toward evidence integration and quality assessment (21, 22, 57).

The analyses of co-cited journals and co-cited authors showed that the major knowledge sources of this field include not only TCM and complementary and alternative medicine journals, but also journals in stroke medicine, neuroscience, and general medicine. Meanwhile, highly influential co-cited authors were mainly scholars specializing in VaD diagnostic criteria, epidemiology, and pathophysiological mechanisms. These findings suggest that research on TCM treatment of VaD does not operate in isolation within its own theoretical system. Instead, it has been gradually embedded within the international mainstream framework of VaD research, continuously absorbing disease definitions, risk-factor frameworks, pathological mechanisms, and clinical endpoints from international VaD studies. In other words, an important premise for the mechanistic deepening of TCM research is that its research topics have gradually been integrated into the common problem structure of modern cerebrovascular disease and cognitive impairment research.

Nevertheless, current herbal medicine research still shows a relatively clear pattern of “strong early-stage evidence but weak downstream translation.” Considerable evidence has accumulated around animal models, histopathology, oxidative stress, inflammatory factors, apoptotic proteins, and signaling pathways (22, 35, 61, 62). However, these mechanistic findings have not yet been fully translated into a stable clinical evidence chain. Many studies have shown that certain formulas, active compounds, or plant-derived components can regulate several molecular indicators and improve cognitive performance in animal models. However, there is still insufficient consistent evidence regarding which mechanistic changes are most closely associated with clinical benefit, which components are the key pharmacodynamic substances, and whether patients with different pathological stratifications derive differential benefits (21, 39). In other words, the main challenge in herbal medicine research is no longer simply whether mechanisms exist, but how to identify, among numerous reported mechanisms, the core mechanisms with genuine disease explanatory power and clinical translational value.

Furthermore, the ongoing debate regarding the quantitative association and causal attribution between VaD and CSVD also suggests that future research in this field should not remain limited to the accumulation of therapeutic evidence under the broad label of VaD. Greater attention should be paid to pathological substrates and phenotypic stratification (58). For example, some study populations may essentially belong to the VCI/CSVD spectrum characterized by cerebral small vessel disease, white matter injury, and chronic hypoperfusion, whereas others may also have prominent Alzheimer’s disease-like pathological changes. Without distinguishing these subtypes (34), mechanistic studies or efficacy evaluations conducted under the generalized concept of VaD may weaken the interpretability of findings and limit the precise definition of TCM therapeutic targets and applicable patient populations. Therefore, future studies should not only continue to deepen mechanistic exploration of inflammation, oxidative stress, and neural repair, but also promote integration among disease concepts, imaging biomarkers, pathological stratification, and intervention research. In this way, research on TCM treatment of VaD can truly move from a “disease-name-oriented” approach toward a “pathological-mechanism-oriented” and “intervenable-link-oriented” research paradigm (59).

### 4.5 Research hotspots and development trends

#### 4.5.1 Herbal medicine research

Among the various intervention modalities used in TCM treatment of VaD, herbal medicine-related research is the most representative and constitutes a major line of continuous knowledge accumulation in the field (22, 60). On the one hand, herbal medicine is one of the most traditional and central forms of TCM intervention for vascular dementia. It includes classical formulas and single herbs, and further extends to active compounds, natural products, and plant-derived nutrients. Therefore, it has a natural translational continuum from traditional theory to modern pharmacology and component characterization (22, 54, 60). On the other hand, based on the keyword distribution, clustering structure, and frontier evolution identified in this study, apart from distinctive branches such as acupuncture, a large body of research focusing on oxidative stress, inflammation, apoptosis, autophagy, chronic cerebral hypoperfusion, and neuroprotection has primarily been advanced through herbal formulas, single herbs, or their natural active components (35, 61, 62). This suggests that, within the research landscape of TCM treatment for VaD, herbal medicine is not merely one intervention modality, but also a key vehicle linking traditional TCM theory, modern mechanistic research, and potential translational application.

For this reason, the distribution of hotspots in herbal medicine research often best reflects changes in the research paradigm of this field. The keyword and clustering results of this study showed that current herbal medicine-related research is not evenly distributed, but is more concentrated in pathological mechanism studies involving “oxidative stress,” “apoptosis,” “inflammation,” and “chronic cerebral hypoperfusion,” showing a certain internal hierarchy. This emphasis on oxidative stress is also consistent with broader food–medicine homology research, in which antioxidant-rich medicinal and edible plants, such as *Phyllanthus emblica* Linn., have been discussed in relation to oxidative stress-associated aging and functional protection; however, such evidence should be interpreted as contextual support for plant-derived antioxidant research rather than direct evidence for VaD treatment (63). Chronic cerebral hypoperfusion can be regarded as the starting point, corresponding to one of the most common pathological backgrounds of VaD. Subsequent processes such as oxidative stress, inflammation, apoptosis, and autophagy imbalance can be understood as secondary injury responses under sustained hypoperfusion. Keywords such as “hippocampus,” “memory,” and “cognitive impairment” more often correspond to the final structural and functional outcomes. This reflects a relatively clear logical chain in current herbal medicine research: beginning with abnormal cerebral perfusion and brain tissue injury, progressing through intermediate mechanisms such as oxidative stress, inflammation, and cell fate regulation, and ultimately leading to improvement in cognitive and behavioral outcomes (35, 61, 62).

Compared with early studies that focused mainly on whether an intervention was effective, this chain-based research design has substantially enhanced the interpretability of mechanistic studies in TCM. It also makes it possible to compare and integrate different herbs, active compounds, and formulas within a shared pathological framework (54, 64). Herbal medicine research has gradually been stratified into several levels, including formulas, single herbs, active compounds, and plant-derived nutrients or natural products. Formula-based research emphasizes multi-component synergy and holistic regulation, which is well suited to the complex pathological network of VaD. Single-herb studies are more conducive to identifying core pharmacodynamic substances and major directions of action. Research on active compounds and plant-derived nutrients is more easily connected with modern pharmacology, molecular mechanisms, and component characterization technologies, thereby improving standardization and international recognition (54, 64). This stratified research framework suggests that the field is attempting to address a key issue in TCM research: how to establish a verifiable, comparable, and translatable framework for explaining therapeutic effects while preserving the advantages of holistic regulation.

This also explains why, in the present study, herbal medicine-related hotspots were connected both to classical pathological mechanisms and to emerging keywords such as “network pharmacology,” “HPLC,” and “expression.” The former indicates that herbal medicine research is actively aligning with mainstream issues in modern VaD research, whereas the latter suggests that researchers have moved beyond demonstrating “efficacy” at the phenomenological level and are now attempting to answer more specific questions: which components exert therapeutic effects, how these components jointly act on multiple pathological nodes, and which molecular changes are merely accompanying phenomena.

Nevertheless, current herbal medicine research still shows a relatively clear pattern of “strong early-stage evidence but weak downstream translation.” Considerable evidence has accumulated around animal models, histopathology, oxidative stress, inflammatory factors, apoptotic proteins, and signaling pathways (22, 35, 35, 62). However, these mechanistic findings have not yet been fully translated into a stable clinical evidence chain. Many studies have shown that certain formulas, active compounds, or plant-derived components can regulate several molecular indicators and improve cognitive performance in animal models. However, there is still insufficient consistent evidence regarding which mechanistic changes are most closely associated with clinical benefit, which components are the key pharmacodynamic substances, and whether patients with different pathological stratifications derive differential benefits (21, 39). In other words, the main challenge in herbal medicine research is no longer simply whether mechanisms exist, but how to identify, among numerous reported mechanisms, the core mechanisms with genuine disease explanatory power and clinical translational value.

Therefore, the future development of herbal medicine research should not merely continue to accumulate conventional indicators such as oxidative stress, inflammation, and apoptosis. Instead, on the basis of existing work, it should further promote external validation that connects intramodel mechanistic loops with clinical endpoints, imaging changes, and pathological classification (13, 39, 64). Only in this way can herbal medicine research truly move from “numerous hotspots” to “stronger explanatory power,” thereby laying a solid foundation for subsequent component development, precision application, and high-quality clinical translation. The herbal and natural-product branch identified in this bibliometric analysis also has implications for the emerging fields of food–medicine homology and neurological nutrition. Herbal formulas, single herbs, natural products, and plant-derived active components may contain polyphenols, flavonoids, terpenoids, alkaloids, polysaccharides, and other bioactive constituents, some of which are derived from medicinal and edible plants. Their reported antioxidant, anti-inflammatory, vascular-protective, metabolic-regulatory, and neuroprotective activities provide a potential conceptual bridge between traditional herbal medicine, functional dietary constituents, and neurovascular health. This connection is particularly relevant to VaD because chronic cerebral hypoperfusion, endothelial dysfunction, blood–brain barrier injury, neuroinflammation, and oxidative stress constitute interconnected pathological processes. However, food–medicine homology should be regarded as a translational perspective for interpreting selected herbal and natural-product studies rather than as a general label for the entire TCM-VaD literature.

#### 4.5.2 Acupuncture research

The results showed that acupuncture is one of the most active and distinctive research branches in this field (42). The frequency of the keyword “acupuncture” was second only to that of “vascular dementia.” As a common complementary intervention in TCM research on VaD, acupuncture-related themes were not scattered randomly across keyword clusters and frontier evolution maps. Instead, they formed a relatively concentrated association structure with terms such as “hippocampus,” “dentate gyrus,” “stimulation,” “activation,” “neural regeneration,” “blood flow,” “hypoperfusion,” “occlusion,” “memory,” and “moxibustion.” This suggests that acupuncture research in VaD has gradually developed a distinctive research pathway centered on regulation of cerebral blood flow, activation of neural function, improvement of hippocampal plasticity, and reconstruction of neural repair processes (37, 38, 65).

This structural feature deserves particular attention. Unlike herbal medicine research, which focuses more on oxidative stress, inflammation, apoptosis, autophagy, and signaling pathway regulation, acupuncture research more clearly points toward “functional regulation and network remodeling.” In particular, the repeated occurrence of keywords such as “hippocampus” and “dentate gyrus” indicates that acupuncture research is not limited to suppressing injury at the molecular level. Rather, it also explores whether acupuncture can promote functional recovery in learning- and memory-related brain regions, improve synaptic plasticity, and even facilitate neuroregeneration (37, 65, 66). The uniqueness of acupuncture in VaD research is increasingly recognized as an intervention acting on the process of brain functional reconstruction, rather than merely as a symptomatic treatment in the conventional sense.

Further integration of keywords such as “blood flow,” “hypoperfusion,” and “occlusion” reveals another important feature of acupuncture research: its research logic often spans both the upstream and downstream ends of the VaD pathological chain. On the one hand, acupuncture has been used to explain effects on cerebral blood perfusion, microcirculatory status, and ischemia-related brain tissue injury. On the other hand, studies further focus on downstream outcomes such as hippocampal function, memory behavior, neural plasticity, and neural repair (37, 65–67). Thus, acupuncture can connect upstream vascular pathological substrates, including cerebrovascular dysfunction, chronic cerebral hypoperfusion, and neurovascular unit imbalance, with downstream neural functional outcomes, including cognitive impairment, hippocampus-dependent learning and memory deficits, and brain functional remodeling. Therefore, the high level of activity in acupuncture research in this field is not merely due to its relative ease of standardization or experimental operation, but also because its mechanisms closely correspond to the continuous pathological chain from impaired blood flow to functional damage in VaD (37, 66, 67).

From a methodological perspective, the relatively dense body of acupuncture-related literature is also related to the intervention characteristics of acupuncture itself. Compared with herbal medicine research, which often faces challenges such as complex components, unclear pharmacodynamic substances, and difficulties in quality control, acupuncture stimulation sites, stimulation methods, frequency, and treatment duration are more easily operationalized and repeated under experimental conditions. Therefore, acupuncture can be more readily integrated with animal models, brain-region observation, molecular biomarker detection, and behavioral assessment (37, 38). This has enabled acupuncture research in VaD to establish a relatively clear research paradigm at an early stage: scalp acupuncture or body acupuncture is applied to ischemia- or hypoperfusion-related models, and its neuroprotective and functional effects are then evaluated using indicators such as hippocampal neuronal injury, neurotransmitter changes, synaptic protein expression, inflammatory factor levels, and behavioral performance. For this reason, acupuncture research may accumulate mechanistic evidence more readily than other forms of TCM intervention, which partly explains its prominent position in the keyword network. Nevertheless, it should also be noted that although acupuncture has accumulated substantial evidence from animal experiments and mechanistic studies, the level and consistency of clinical evidence still need to be improved, particularly in terms of high-quality randomized controlled trials, unified outcome measures, and long-term follow-up assessments (68–70).

#### 4.5.3 Integration of frontier technologies

In recent years, keywords such as “network pharmacology,” “HPLC,” “expression,” “inflammasome,” and “alpha-synuclein” have gradually appeared as emerging topics, suggesting that research on TCM treatment of VaD is increasingly incorporating component analysis, computational prediction, expression profiling, and neuroinflammation-related concepts. However, these burst terms should be interpreted as indicators of increasing methodological attention rather than direct evidence of validated mechanistic progress. Network pharmacology, in particular, can help generate hypotheses about potential “component–target–pathway–phenotype” relationships, but such predictions remain insufficient without experimental confirmation of chemical constituents, pharmacologically relevant exposure, target engagement, dose–response relationships, and causal pathway validation (22, 54). Therefore, the value of these frontier technologies lies not in proving mechanisms by themselves, but in helping researchers generate testable hypotheses and prioritize candidate mechanisms for further validation.

From the keyword structure revealed by the results, the increasing activity of analytical techniques such as HPLC/UPLC indicates that researchers have begun to place greater emphasis on the material basis and quality control of TCM formulas, single herbs, and natural products, aiming to transform complex and heterogeneous interventions into more identifiable and comparable chemical entities (71, 72). The emergence of “network pharmacology” suggests that, after partially clarifying chemical components, researchers have further attempted to establish potential links among components, targets, pathways, and phenotypes, in order to explain the multi-component and multi-target action patterns of TCM (54, 73). The burst keyword “expression” further indicates that the research paradigm in this field is gradually shifting from the traditional approach of prespecifying and validating pathways to a new approach that uses high-dimensional observation of global molecular responses to infer key mechanistic modules (54, 72). Collectively, these frontier terms do not simply indicate replacement of one technique by another; rather, they point to a transformation of research logic from low-dimensional validation to high-dimensional screening and, ultimately, systems-level integration.

These keywords suggest that the field has begun to move beyond a fixed reliance on a small number of classical inflammatory factors, apoptotic proteins, or oxidative stress indicators, and is instead attempting to identify broader changes in molecular expression profiles before and after intervention (54, 72). VaD itself is a highly heterogeneous disease, with pathological substrates involving chronic hypoperfusion, white matter injury, neuroinflammation, metabolic imbalance, neurovascular unit dysfunction, and potential overlap with neurodegenerative pathology. If research remains limited to a single pathway or a few biomarkers, it may easily overstate the general importance of local pathway changes. In contrast, the rise of expression profile-related studies has encouraged the field to identify, from a more open molecular perspective, which changes are shared, which are specific to certain pathological backgrounds, and which may represent true hub nodes.

For this reason, the natural extension of burst terms such as “expression” is not simply “measuring more genes or proteins,” but rather the increasing rationale for the combined application of multi-omics. Specifically, transcriptomics can help reveal the global changes in gene programs related to cellular stress, inflammatory cascades, metabolic reprogramming, and synaptic plasticity after TCM intervention. Proteomics can further determine whether these transcriptional changes are translated into functional proteins and signaling pathways, making it particularly suitable for identifying key modules such as inflammatory amplification, mitochondrial dysfunction, cytoskeletal remodeling, and synaptic protein damage. Metabolomics is more suitable for investigating energy metabolism disorders, lipid metabolic disturbances, redox imbalance, and gut–brain axis-related metabolic alterations, all of which are important in VaD but difficult to explain using a single indicator (54, 71, 72). For TCM interventions, this combined multi-omics approach has particular methodological advantages, because TCM itself is a typical multi-component and multilevel intervention. Its therapeutic effects may not necessarily be reflected in regulation of a single pathway, but rather in the coordinated correction of multiple molecular networks and metabolic pathways (54, 72).

Furthermore, combining HPLC/UPLC and network pharmacology with multi-omics may help establish a more complete mechanistic analysis pathway than traditional approaches. First, component analysis can be used to identify relatively stable and traceable candidate components in formulas or natural products. Then, network pharmacology can be applied to preliminarily screen potential targets and pathways. Subsequently, transcriptomics, proteomics, and metabolomics can be used to observe the actual molecular responses occurring after intervention. Finally, functional experiments can verify the necessity and causality of key nodes (54, 71–73). Beyond conventional cell and animal experiments, organ-on-a-chip technology has also been proposed as a promising platform for natural product evaluation, because it may provide more physiologically relevant microenvironments for assessing efficacy, toxicity, and mechanistic responses, thereby offering a potential complementary validation tool for future TCM-related mechanistic studies (74). In this way, research is no longer limited to the static speculation that “a given formula may act on many targets,” but can gradually develop into dynamic evidence integration that clarifies which candidate components correspond to which real molecular changes, and which of these changes are genuinely associated with pathological improvement. For a disease such as VaD, in which pathological processes are highly interconnected, this continuous evidence chain from chemical components to molecular networks and then to functional outcomes is clearly more explanatory than simply listing several related pathways (75).

On this basis, the potential value of spatial omics deserves particular forward-looking discussion. Although spatial transcriptomics and spatial proteomics have not yet become high-frequency keywords in this study, the research directions indicated by keywords such as “expression,” “inflammasome,” “hippocampus,” “dentate gyrus,” and “chronic cerebral hypoperfusion” suggest that these technologies may become important extensions in the next stage (76, 77). The reason is that the pathophysiological processes of VaD are highly spatiotemporally specific. The key question is not only “which molecules change,” but also “in which cell types, brain regions, microenvironments, and neurovascular interfaces these changes occur.” For example, is inflammasome activation mainly concentrated in microglia, endothelial cells, or astrocytes? Are molecular responses in the hippocampus, white matter, and cortex consistent under chronic hypoperfusion? Do certain active components of TCM exert spatially heterogeneous effects on neurons, vascular endothelial cells, and glial cells? These questions are difficult to answer clearly using only bulk tissue homogenate assays. Spatial transcriptomics or spatial proteomics can provide integrated information linking molecular changes, cell types, and tissue localization (76, 77). Therefore, spatial multi-omics can help the field move from “knowing that a pathway has changed” to “knowing at which interface the change occurs, which cell population drives it, and whether it corresponds to local pathological injury.”

Similarly, the emergence of “inflammasome” and “alpha-synuclein” provides clear problem-oriented directions for multi-omics and spatial analysis. The former suggests that inflammation research is shifting from general inflammatory factor changes toward identification of key amplification hubs and intercellular inflammatory communication networks (78). The latter indicates that the research boundary is beginning to extend from purely vascular injury to the intersection of mixed dementia and protein pathology (79). In this context, single-level biomarker detection is increasingly insufficient to fully explain the disease process. In contrast, if inflammatory responses, metabolic remodeling, proteostasis imbalance, and intercellular interactions can be observed simultaneously at the omics level, and if their spatial distribution in the hippocampus, white matter, cerebral microvessels, and neurovascular unit can be identified, it may become more possible to explain why TCM can exert “multi-target but not centerless” effects under complex pathological conditions. In other words, these frontier keywords themselves indicate that the problems facing this field are becoming more complex, and this complexity requires a methodological shift from single-dimensional validation to multidimensional coupling.

Of course, it should be emphasized that the introduction of multi-omics and spatial omics does not automatically make a study more “advanced” or its conclusions more reliable. Whether network pharmacology, transcriptomics, proteomics, metabolomics, or spatial omics is used, these methods essentially serve as tools to expand observational dimensions, generate mechanistic hypotheses, and screen key nodes; they do not automatically provide causal evidence (72, 73, 76, 77, 79). This is especially important in TCM research. Without reasonable component control, stratification of pathological models, interventional validation of key nodes, and correspondence with behavioral or imaging endpoints, even highly complex omics data may remain merely “information-rich but interpretively diffuse.” Therefore, the key to future integration of frontier technologies should not be the simple accumulation of data types, but rather a stricter research logic: starting from a clearly defined pathological question, building on component analysis, using multi-omics screening as a tool, supporting findings with spatial localization and functional validation, and ultimately returning to disease stratification and clinical translatability (54, 71–73, 76, 77, 79).

#### 4.5.4 An integrative perspective centered on the blood–brain barrier

Notably, many high-frequency hotspots and frontier themes identified in this study, such as oxidative stress, inflammation, apoptosis, chronic cerebral hypoperfusion, cerebral blood flow disturbance, and neuroregeneration, correspond to pathological processes at different levels. However, from the perspective of VaD pathogenesis, these changes can be integrated at a higher level into a shared framework of blood–brain barrier (BBB) and neurovascular unit (NVU) injury (13, 80–83). Increasing evidence suggests that the BBB is not merely a passive barrier, but a dynamic interface that maintains brain microenvironmental homeostasis, couples cerebral blood flow regulation with neural activity, and participates in immune surveillance and metabolite transport. When its structure and function are impaired, cascade dysregulation may occur across endothelial cells, pericytes, astrocytes, and the basement membrane, thereby amplifying the onset and progression of cognitive impairment (13, 81, 83).

In the context of VaD/VCI, chronic cerebral hypoperfusion and cerebral small vessel disease are widely regarded as important upstream events driving BBB/NVU imbalance. Sustained hypoperfusion can lead to cerebral microvascular endothelial dysfunction, disruption of tight junction proteins, insufficient white matter perfusion, and increased brain barrier permeability. Subsequently, plasma protein extravasation, inflammatory cell recruitment, oxidative stress amplification, mitochondrial dysfunction, and myelin/axonal injury may mutually reinforce one another, ultimately leading to white matter lesions, hippocampal and subcortical network damage, and cognitive decline (13, 80, 82, 84, 85). Therefore, from a pathophysiological perspective, the seemingly scattered hotspots identified in this study—such as oxidative stress, inflammation, hypoperfusion, cell death, and neural repair—are not isolated processes, but may converge on the central pathological link of BBB disruption and NVU homeostatic imbalance.

Furthermore, the integrative value of the BBB perspective lies in its ability to place vascular and neural factors within a shared interface. Traditionally, oxidative stress, inflammation, apoptosis, and neuroregeneration are often discussed as separate mechanistic modules. However, within the BBB/NVU framework, these processes can be understood both as causes of barrier injury and as amplified consequences of barrier disruption. For example, inflammasome activation and abnormal communication among microglia, endothelial cells, and astrocytes may not only aggravate local inflammatory cascades, but also further alter cerebral blood flow regulation, transport function, and local microenvironmental homeostasis, thereby making vulnerable regions such as white matter and the hippocampus more susceptible to injury (81–83, 86). This also means that the BBB is not merely a morphological structure, but an important pathological interface linking microcirculatory disturbance, inflammatory amplification, metabolic dysregulation, and neural functional outcomes.

From the perspective of TCM research, the BBB/NVU framework is particularly informative. Previous studies have found that certain phytochemicals and active components of Chinese medicines may interfere with the progression of VCI/VaD through multiple pathways, including maintaining BBB structural integrity, regulating tight junction protein expression, suppressing neuroinflammation, reducing oxidative stress, improving microcirculation, and modulating the gut–brain axis (87–90). This suggests that the hotspots identified in the present bibliometric analysis, including inflammation, oxidative stress, chronic hypoperfusion, and neural repair, are not merely scattered mechanistic labels. Instead, they may collectively point to a more integrative pathological core of TCM intervention in VaD: maintenance of BBB and NVU homeostasis (87–90). Especially given the multi-component and multi-target features of TCM, its effects may not be limited to inhibition of a single inflammatory factor or change in an antioxidant indicator, but may be more likely reflected in coordinated correction of the “microvasculature–barrier–glia–neuron” pathological unit (91). This view is compatible with the broader food–medicine homology literature, which increasingly emphasizes functional components, bioactive constituents, and multi-level biological regulation rather than single-compound explanations alone (23, 24).

Based on this perspective, future research on TCM treatment of VaD may further shift from “single-pathway validation” toward “key-interface integration.” In particular, against the background of continued advances in network pharmacology, expression profiling, and component detection technologies, integrating active component screening, molecular mechanism studies, BBB functional assessment, NVU injury repair, region-specific changes in white matter and the hippocampus, and cognitive behavioral improvement may help enhance the systematic nature and mechanistic explanatory power of the field (82, 83, 87, 88). In addition, with the development of fluid biomarkers of BBB/NVU injury, neuroimaging, and spatial omics, future studies may more precisely identify dominant types of barrier injury in patients with different pathological strata, thereby providing stronger support for target focusing and population definition in TCM intervention (86, 92).

Of course, it should be noted that the BBB, as an integrative framework, is primarily a forward-looking inference based on the hotspot distribution identified in this study and previous mechanistic research. It cannot replace direct high-quality experimental and clinical evidence. Its true value still depends on further validation based on component control, pathological model stratification, and correspondence with clinical endpoints.

### 4.6 Clinical evidence and translational considerations

Although the bibliometric results identified “efficacy,” “therapy,” “trial,” “protocol,” and “safety” as recurring clinical keywords, these terms should not be interpreted as demonstrating strong clinical evidence by themselves. The clinical relevance of TCM-related interventions for VaD remains closely dependent on the quality and reproducibility of the underlying clinical studies. First, diagnostic heterogeneity is a major challenge. VaD may be defined using different diagnostic criteria, and some studies may include patients with broader vascular cognitive impairment, post-stroke cognitive impairment, cerebral small vessel disease-related cognitive decline, or mixed vascular–neurodegenerative pathology. Such heterogeneity may weaken the comparability of clinical findings and obscure which patient subgroups are most likely to benefit from specific TCM-related interventions.

Second, many clinical studies in this field may be limited by small sample sizes, single-center designs, short treatment courses, and insufficient follow-up duration. These limitations make it difficult to determine whether observed cognitive improvements are stable, clinically meaningful, or generalizable. Outcome assessment also requires greater standardization. Cognitive scales such as MMSE, MoCA, ADAS-Cog, activities of daily living, global clinical impression, neuropsychiatric symptoms, and caregiver-related outcomes may capture different dimensions of VaD-related impairment. Future trials should predefine primary and secondary outcomes, incorporate functional and quality-of-life endpoints, and consider imaging or biomarker-based stratification when appropriate.

Third, intervention standardization is particularly important for both TCM formulas and acupuncture-related studies. For herbal formulas, future studies should clearly report prescription composition, herb dosage, preparation method, quality control, treatment duration, concomitant medication, and the role of syndrome differentiation. Syndrome differentiation is central to TCM practice, but if it is not transparently defined and operationalized, it may reduce reproducibility and complicate comparison across trials. For acupuncture, electroacupuncture, and moxibustion studies, detailed reporting of acupoint selection, stimulation intensity, needle retention time, frequency, treatment course, practitioner expertise, and sham or active comparator design is essential. Because blinding is difficult in non-pharmacological interventions, studies should at least strengthen assessor blinding, allocation concealment, and objective outcome evaluation where possible.

Finally, comparator selection and safety reporting need greater attention. Comparators may include usual care, placebo or sham intervention, active drugs, rehabilitation, cognitive training, or combined therapy, and these choices directly affect interpretation of treatment effects. Safety reporting should include adverse events related to herbal medicines, herb–drug interactions, acupuncture-related events, moxibustion-related burns or discomfort, dropout reasons, and long-term tolerability. Therefore, future clinical research should move beyond reporting whether an intervention is “effective” and should instead emphasize rigorous diagnostic classification, standardized intervention reporting, adequate sample size, appropriate controls, blinded outcome assessment, longer follow-up, and transparent safety evaluation. These improvements are essential for translating bibliometric hotspots into clinically meaningful and reproducible evidence.

### 4.7 Animal model validity and translational limitations

Animal models constitute an important foundation for mechanistic studies of TCM-related interventions for VaD, but their translational limitations should be carefully considered. The present bibliometric results showed frequent attention to chronic cerebral hypoperfusion, rat models, bilateral common carotid artery occlusion, oxidative stress, inflammation, apoptosis, autophagy, and hippocampal injury. These findings suggest that a substantial portion of the mechanistic literature is organized around hypoperfusion- and occlusion-based preclinical models. Such models are useful for investigating cerebral blood-flow reduction, ischemic neuronal injury, oxidative stress, microglial activation, hippocampal dysfunction, and cognitive impairment, and they provide relatively reproducible platforms for evaluating herbal formulas, natural products, acupuncture, electroacupuncture, and moxibustion.

However, these models do not fully reproduce the clinical heterogeneity of human VaD and the broader VCI spectrum. Human vascular cognitive impairment often develops in the context of ageing, hypertension, diabetes, dyslipidemia, cerebral small vessel disease, endothelial dysfunction, blood–brain barrier disruption, white matter lesions, lacunes, strategic infarcts, post-stroke cognitive impairment, and mixed Alzheimer-related pathology. In contrast, commonly used bilateral common carotid artery occlusion or chronic hypoperfusion models usually emphasize reduced cerebral perfusion and downstream hippocampal or neuronal injury. As a result, the bibliometric prominence of oxidative stress, apoptosis, hippocampal injury, and hypoperfusion-related mechanisms may partly reflect model dominance rather than the full biological complexity of clinical VaD.

This model dependence has important implications for interpreting TCM-related mechanisms. For example, an intervention that improves cognitive behavior or reduces oxidative stress in a hypoperfusion model may not necessarily show the same effect in patients with mixed vascular–neurodegenerative pathology, extensive white matter disease, metabolic comorbidities, or post-stroke cognitive impairment. Similarly, acupuncture- or moxibustion-related effects on cerebral blood flow in experimental occlusion models may not directly translate to patients whose cognitive decline is driven by diffuse microvascular injury or chronic neurodegenerative comorbidity. Therefore, future studies should diversify animal models and incorporate aged animals, vascular risk-factor models, small-vessel disease models, white matter injury models, post-stroke cognitive impairment models, and mixed pathology models when appropriate.

In addition, preclinical studies should strengthen translational design by linking behavioral outcomes with imaging, histopathology, vascular function, blood–brain barrier integrity, white matter injury, synaptic markers, and neuroinflammatory or metabolic indicators. Model selection should be explicitly aligned with the clinical subtype or pathological process being targeted. Such improvements would help determine whether the mechanisms identified in animal studies represent generalizable VaD biology or model-specific injury responses.

### 4.8 Limitations

Although this study provides a systematic bibliometric overview of publication trends, knowledge structures, and emerging themes in TCM-related research on VaD, several limitations should be acknowledged.

A major limitation concerns the data source. This study used only the Web of Science Core Collection (WoSCC) for bibliometric analysis. Although WoSCC provides standardized citation information and is widely used in bibliometric studies, using a single database may have limited literature coverage and affected the observed country distribution, institutional rankings, author collaboration networks, journal distribution, keyword frequencies, and citation patterns. This limitation is particularly relevant for a TCM-focused topic, because important studies may also be indexed in Scopus, PubMed/MEDLINE, Embase, CNKI, Wanfang, VIP, or SinoMed. The omission of Chinese-language databases may have led to underrepresentation of clinical experience, formula-based studies, acupuncture research, and other locally published TCM evidence. Therefore, the findings of this study should be interpreted as reflecting the WoSCC-indexed international literature rather than the entire academic landscape of TCM treatment for VaD.

The English-language restriction may also have introduced language bias. Because many clinical and experimental studies on TCM are published in Chinese, excluding non-English records may underestimate the size, diversity, and clinical characteristics of this field. This restriction may also influence the interpretation of country, institution, author, and intervention patterns, particularly the apparent dominance of China in the English-indexed literature.

The search strategy was designed to balance sensitivity and specificity, but retrieval bias cannot be completely avoided. The TCM-related terms included broad expressions such as “natural product,” “natural compound,” “phytochemical,” “secondary metabolite,” “natural medicine,” and “herb,” which helped capture studies that may not be indexed explicitly under “traditional Chinese medicine.” However, these broad terms may have retrieved some studies that were not strictly rooted in TCM theory or classical Chinese herbal medicine. Conversely, because the disease-related search strategy was centered on VaD-related terms, relevant studies indexed primarily under VCI, VCID, post-stroke cognitive impairment, cerebral small vessel disease, chronic cerebral hypoperfusion, or mixed dementia may have been missed. Although manual screening was performed to improve thematic relevance, potential false-positive and false-negative records cannot be completely excluded.

Another important issue is intervention heterogeneity. The included literature covers Chinese herbal formulas, single herbs, herbal extracts, natural products, isolated active compounds, acupuncture, electroacupuncture, moxibustion, and mixed or unspecified TCM-related interventions. These categories differ markedly in theoretical basis, biological mechanism, clinical implementation, standardization requirements, and evidence level. Therefore, the bibliometric findings should not be interpreted as applying equally to all TCM-related interventions. In particular, conclusions regarding food–medicine homologous resources, natural products, or phytoactive compounds mainly apply to the herbal and natural-product branch and should not be generalized to acupuncture- or moxibustion-related studies without subgroup-specific evidence. Moreover, food–medicine homology was introduced as an interpretive framework rather than as a predefined retrieval domain; therefore, the present analysis cannot be considered a comprehensive bibliometric mapping of literature specifically focused on medicinal and edible plant resources.

Citation-based indicators should also be interpreted cautiously. Total citations, average citations, co-citation frequency, betweenness centrality, and burst strength may be influenced by publication year, journal visibility, database coverage, language restriction, disciplinary citation practices, author networks, and document type. Thus, citation frequency or network centrality should be understood as indicators of bibliometric visibility, intellectual linkage, or research attention, rather than as direct evidence of methodological quality, clinical importance, therapeutic efficacy, safety, or mechanistic validity. Temporal bias should also be considered, because older publications have had more time to accumulate citations, whereas newly emerging topics may not yet have stable citation patterns.

Keyword based analyses are similarly affected by metadata quality and term standardization. Although keyword cleaning and harmonization were performed differences in spelling abbreviations synonym merging database indexing practices author-selected keywords and possible metadata errors may still influence keyword frequency cluster structure and burst detection. Some broad terms such as “brain,” “model,” “mechanisms,” and “impairment,” were retained for transparency when they met the frequency threshold but they should not be overinterpreted as independent research themes.

Most importantly, bibliometric analysis is a science-mapping method and cannot replace systematic evidence appraisal. This study did not assess risk of bias, certainty of evidence, intervention effect size, clinical efficacy, safety, adverse events, or clinical applicability. Therefore, the identified hotspots and emerging themes should be interpreted as research trends and hypothesis-generating directions rather than validated therapeutic conclusions. Future studies should combine bibliometric mapping with systematic reviews, risk-of-bias assessment, meta-analysis where appropriate, mechanistic validation, and well-designed clinical trials to determine the true efficacy, safety, and translational value of TCM-related interventions for VaD.

## 5 Conclusion

Based on bibliometric methods, this study systematically revealed the research landscape, hotspot themes, and evolutionary trends in the field of TCM treatment for VaD from 1997 to 2025. Overall, the field has progressed from early exploration to sustained publication activity and increasingly diverse research topics. China was the largest contributor in terms of publication output and occupied a central position in the institutional and collaboration networks. However, this pattern should be interpreted in light of database selection, English-language indexing, TCM specialization, domestic research networks, and disciplinary citation practices. There remains room for improvement in the breadth of international collaboration and the production of methodologically rigorous and internationally representative studies.

The results showed that the research focus of TCM treatment for VaD has gradually shifted from early efficacy observation toward mechanism-related interpretation and technology-assisted analysis. Current hotspots are distributed across two major intervention branches. The herbal and natural-product branch includes herbal formulas, single herbs, natural products, active compounds, and plant-derived constituents, and is mainly associated with oxidative stress, inflammation, apoptosis, autophagy, chronic cerebral hypoperfusion, component characterization, and neurovascular protection. In contrast, acupuncture, electroacupuncture, and moxibustion are more closely related to neuromodulation, hippocampal plasticity, cerebral perfusion, and functional recovery. Within the former branch, selected medicinal and edible plant resources and phytoactive compounds provide a relevant interface with food–medicine homology and neurological nutrition; however, this perspective should not be generalized to the entire TCM-VaD literature. Meanwhile, the emergence of frontier themes such as “network pharmacology,” “HPLC,” “expression,” “inflammasome,” and “alpha-synuclein” indicates increasing bibliometric attention to material basis characterization, molecular mechanism exploration, and the combined application of multiple technologies. These findings describe research trends within the literature and should not be regarded as direct evidence of methodological rigor, therapeutic efficacy, or clinical translational readiness.

In summary, research on TCM treatment of VaD is currently characterized by sustained publication growth, expanding mechanistic topics, methodological diversification, and increasing interest in evidence integration. Future studies should further strengthen disease stratification and standardized diagnosis, improve the quality of basic and clinical research, promote the integration of multi-omics with component-based mechanistic studies, and evaluate the translational value of experimental findings through rigorous preclinical validation and well-designed clinical studies. This study provides a reference for understanding the developmental trajectory of the field, identifying future research directions, and promoting more rigorous research on TCM treatment for VaD.

## Data Availability

The original contributions presented in the study are included in the article/supplementary material, further inquiries can be directed to the corresponding authors.
